# Study of SQ109 analogs binding to mycobacterium MmpL3 transporter using MD simulations and alchemical relative binding free energy calculations

**DOI:** 10.1007/s10822-023-00504-6

**Published:** 2023-05-02

**Authors:** Marianna Stampolaki, Ioannis Stylianakis, Helen I. Zgurskaya, Antonios Kolocouris

**Affiliations:** 1 Laboratory of Medicinal Chemistry, Section of Pharmaceutical Chemistry, Department of Pharmacy, National and Kapodistrian University of Athens, Panepistimiopolis-Zografou, 15771 Athens, Greece; 2 Department of Chemistry and Biochemistry, University of Oklahoma, Stephenson Life Sciences Research Center, 101 Stephenson Parkway, Norman, OK 73019-5251 USA; 3 Department of NMR-Based Structural Biology, Max Planck Institute for Multidisciplinary Sciences, Am Faßberg 11, 37077 Göttingen, Germany

**Keywords:** SQ109, MD simulations, Alchemical relative binding free energy, MM-GBSA, MmpL3, Binding affinities

## Abstract

*N*-geranyl-*N*΄-(2-adamantyl)ethane-1,2-diamine (SQ109) is a tuberculosis drug that has high potency against *Mycobacterium tuberculosis (Mtb)* and may function by blocking cell wall biosynthesis. After the crystal structure of MmpL3 from *Mycobacterium smegmatis* in complex with SQ109 became available, it was suggested that SQ109 inhibits Mmpl3 mycolic acid transporter. Here, we showed using molecular dynamics (MD) simulations that the binding profile of nine SQ109 analogs with inhibitory potency against Mtb and alkyl or aryl adducts at C-2 or C-1 adamantyl carbon to MmpL3 was consistent with the X-ray structure of MmpL3 – SQ109 complex. We showed that rotation of SQ109 around carbon–carbon bond in the monoprotonated ethylenediamine unit favors two *gauche* conformations as minima in water and lipophilic solvent using DFT calculations as well as inside the transporter’s binding area using MD simulations. The binding assays in micelles suggested that the binding affinity of the SQ109 analogs was increased for the larger, more hydrophobic adducts, which was consistent with our results from MD simulations of the SQ109 analogues suggesting that sizeable C-2 adamantyl adducts of SQ109 can fill a lipophilic region between Y257, Y646, F260 and F649 in MmpL3. This was confirmed quantitatively by our calculations of the relative binding free energies using the thermodynamic integration coupled with MD simulations method with a mean assigned error of 0.74 kcal mol^−1^ compared to the experimental values.

**Graphical abstract:**

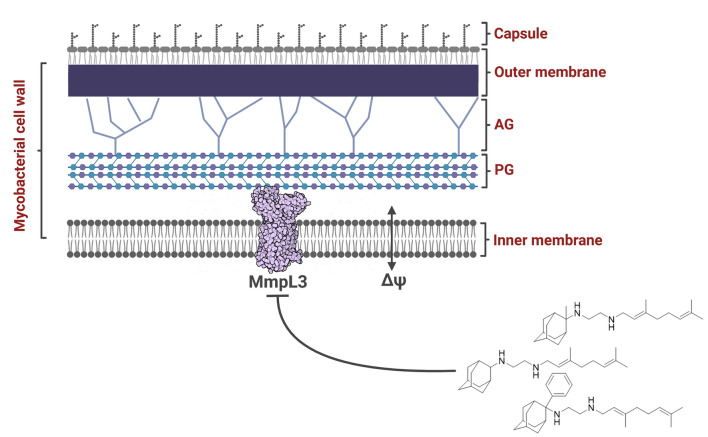

**Supplementary Information:**

The online version contains supplementary material available at 10.1007/s10822-023-00504-6.

## Introduction

**Fig. 1:**
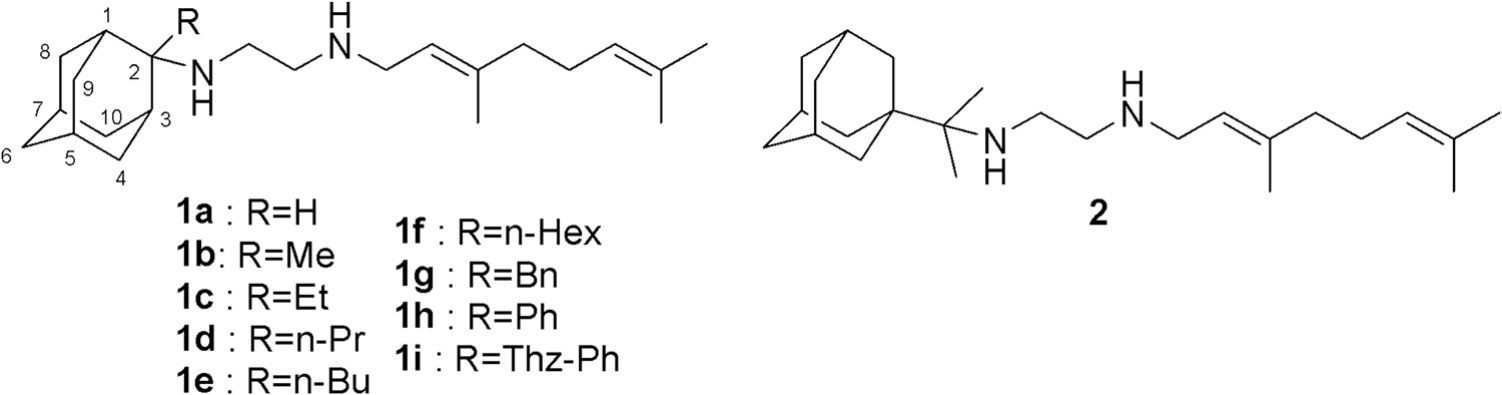
Chemical structures of SQ109 (**1a**) and other ethylenediamine analogs **1b-i** or **2** with adducts at C-1 or C-2 adamantyl carbon, respectively, studied in the present work (Thz-Ph: 5-phenylthiazol-2-yl)

In 2020, an estimated 1.9 million people died from *tuberculosis* (*TB*), the leading cause of death among carriers of HIV [1]. The *N*-geranyl-*N*΄-(2-adamantyl)ethane-1,2-diamine SQ109 (**1a**), [2] shown in Fig. 1, is a second generation ethylenediamine drug, after ethambutol against *Mycobacterium tuberculosis* (*Mtb*). Indeed, SQ109 (**1a**) has been in phase II clinical trials [3, 4] and shows high potency against drug resistant *Mtb* [5–7]. The importance of SQ109 (**1a**) as a highly potent therapeutic agent, triggered the synthesis of analogs [8–13] aiming at the improvement of drug potency, the spectrum of biological activity and the pharmacokinetic properties.

Ethambutol has been suggested to inhibit *Mtb* by binding to the membrane-embedded Emb proteins, EmbB and EmbC, involved in arabinan biosynthesis, [14] while SQ109 (**1a**) has been suggested that targets the trehalose monomycolate transporter, Mycobacterial membrane protein Large 3 (MmpL3), [4, 5] and like ethambutol, inhibits cell wall biosynthesis. MmpL3 is a membrane protein, essential for the translocation of mycolic acids in the form of trehalose monomycolates from their production site in the cytoplasm to the periplasmic space, where mycolic acids can be used in assembly of the *Mtb* outer membrane [15, 16]. Its transporter activity is driven by a proton motive force (PMF) to describe that coupled with the movement of substrates toward the periplasm, protons flow into the cytoplasm to energize this translocation process. Two pairs of D-Y (D256-Y646 and D645-Y257) allow such proton translocation and these D-Y pairs are a conserved feature of the MmpL family of transporters [10, 16–18]. In 2019, the X-ray structure (PDB ID 6AJG [17]) of MmpL3 from *Mycobacterium smegmatis* (*Ms*) in complex with SQ109 (**1a**) or other *Mtb* inhibitors found inside the transporter’s pore as well as the X-ray structure of the MmpL3 – 1,2-dipalmitoyl-*sn*-glycero-3-phosphoethanolamine (POPE) complex (PDB ID 6OR2) [19] became available. Afterwards, structures of the apo-protein [20, 21] or in complex with additional ligands [21, 22] were also reported, using cryo-electron microscopy (cryo-EM) or X-ray crystallography.

The ability of SQ109 (**1a**) to inhibit the function of MmpL3 can be explained on the basis of its direct binding to MmpL3 and disrupting transporter’s proton translocation [17, 23] or by uncoupling activity on the PMF through another mechanism [5, 7, 24, 25] which is not specific to *Mycobacteria* [26]. The latter mechanism of action may be consistent with the broad-spectrum of activity [4] of SQ109 (**1a**) against pathogens that lack MmpL3 [27].

Using multiscale thermophoresis (MST) in native cell membrane nanoparticle environment, a dissociation constant (K_d_) of ~ 1.6 μM for SQ109 (**1a**) has been reported which is consistent with low μΜ potency of SQ109 (**1a**) against *Mtb*, while is much smaller than the ~ 0.4–1.2 mM measured using surface plasmon resonance (SPR) in micelles [17, 28]. In a previous paper [13] we synthesized the SQ109 (**1a**) analogs **1b-i, 2** some of which exhibited low μΜ biological potency against *Mtb* and had mid-micromolar binding affinity to MmpL3 in micelles measured using SPR. While measurement of binding affinity in membranes with MST are more consistent with biological activities of SQ109 (**1a**) and its analogs against *Mtb* relative binding free energies even measured with SPR might provide useful values to compare them with calculated relative binding free energies as model that can support binding of **1b-i, 2** in the same binding site as SQ109 (**1a**) in MmpL3.

Thus, here, we explored the binding profile of our synthesized  SQ109 (**1a**) analogs **1b-g, 2** to MmpL3 by applying: (a) molecular dynamics (MD) simulations (~ 7.2 μs total simulation time) using the X-ray structure of SQ109 (**1a**) with MmpL3 (PDB ID 6AJG [17]) as reference structure; (b) binding free energy calculations with the Molecular Mechanics-Generalized Born Surface Area (MM-GBSA) method; [29–31] (c) alchemical relative binding free energies calculations using the thermodynamic integration combined with MD simulations (TI/MD) method. [32–35] We next compared if the calculated relative binding free energies agreed with experimental relative binding free energies measured previously with SPR against *Mtb* MmpL3 (MtMmpL3) [13].

We explored the conformational properties of SQ109 (**1a**) in solution using Density Functional Theory (DFT) calculations to compare the conformational properties of its analogs **1b-i, 2** in solution and in bound state with MmpL3 described by docking calculations and MD simulations. Thus, we calculated the free energies of the conformational minima of SQ109 (**1a**) by rotation around bonds that involve the ethylenediamine unit (three dihedral angles) in hydrophilic environment or hydrophobic environment using Density Functional Theory (DFT). The DFT calculations showed that in both hydrophilic or hydrophobic environments and inside the transporter’s pore as showed from MD simulations the central ethylenediamine carbon–carbon bond in favored a *gauche* conformation.

Our MD simulations suggested that alkyl or aryl substituents at the adamantyl C-2 of SQ109 (**1a**) can fill the region between Y257, Y646, F260 and F649 in MmpL3 of *Ms* or *Mtb*. The TI/MD calculations of relative binding free energies were  consistent with  the experimental relative binding free energies  measured using SPR and showing that binding strength is increased by increasing the size of the C-2 adamantyl adduct. Overall, the MD simulations combined with TI/MD calculation results suggested that MmpL3 can likely form stable complexes with ligands **1b-i, 2** which bind MmpL3 at the same site with SQ109 (**1a**).

## Results

### Conformational analysis of monoprotonated ethylenediamine unit in SQ109 (1a)

The ethylenediamine unit in SQ109 (**1a**) involves three dihedrals affecting the drug’s orientation inside the MmpL3 pore through rotation around (2-Ad)NHCH_2_–CH_2_NH_2_^+^Ger, (2-Ad)NHCH_2_CH_2_–NH_2_^+^Ger and C_1,Ad_C_2,Ad_–NHCH_2_CH_2_NH_2_^+^Ger bonds (where C1,Ad or C2,Ad are adamantyl carbons at position-1, or -2 in 2-adamantyl group) described in  Tables 1–3. Using the B3LYP functional with dispersion interactions correction (B3LYP-D3) [36–39] and the 6-31G(d,p) basis set calculations we performed full geometry optimization and calculated the free energies of the conformational minima of SQ109 (**1a**), by manual rotation around these three dihedrals in a hydrophilic and a hydrophobic environment. Dispersion correction improves the calculation of the forces acting on the atoms in distances > 3 Å and the accuracy of relative conformational energies calculation which are shown in Table 1. The hydrophilic environment was simulated with an implicit water environment and a dielectric constant (*ε*) = 80 and the hydrophobic environment was simulated with an implicit chloroform environment and *ε* = 4.8 using the polarizable continuum model (PCM) [41] and taking advantage of a smooth switching function [42].

**Table 1:** Calculations of conformational free energies by rotation of (2-Ad)CH_2_–CH_2_NH_2_^+^Ger in SQ109 (**1a**) using the B3LYP-D3 /6-31G(d,p) and PCM for water (*ε* = 80) or chloroform (*ε* = 4.8)


Dielectric	Δ*G* (kcal mol^−1^) for SQ109(**1a**)		
	conformer		
	*gauche*(−)	*gauche*( +)	*anti*
*ε* = 80	2.95	0	10.23
*ε* = 4.8	2.92	0	8.80

**Table 2:** Calculations of conformational energies by rotation of (2-Ad)CH_2_CH_2_–NH_2_^+^Ger in SQ109 (**1a**) using the B3LYP-D3 /6-31G(d,p) and PCM for water (*ε* = 80) or chloroform (*ε* = 4.8)


Dielectric	Δ*G* (kcal mol^−1^) for SQ109(**1a**)		
	Conformer		
	*gauche*(−)	*gauche*( +)	*anti*
*ε* = 80	2.77	2.78	0
*ε* = 4.8	2.85	2.94	0

**Table 3:** Calculations of conformational energies by rotation around C_1,Ad_C_2,Ad_–NHCH_2_ in SQ109 (**1a**) using the B3LYP-D3 /6-31G(d,p) and PCM for water (*ε* = 80) and chloroform (*ε* = 4.8)


dielectric	Δ*G* (kcal mol^−1^) for SQ109(**1a**)		
	Conformer		
	*gauche*(−)	*gauche*( +)	*anti*
*ε* = 80	0	3.69	8.24
*ε* = 4.8	0	3.61	9.25

**Fig. 2:**
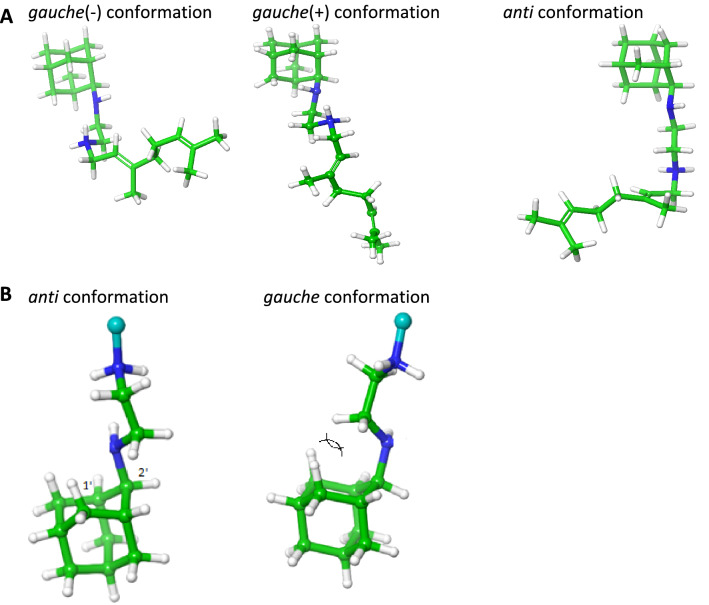
Important conformational features of SQ109 (**1a**). In (A) are shown the conformations *gauche*(-) or *gauche*( +) or *anti* by rotation around the ethylenediamine’s unit carbon–carbon bond, (2-Ad)CH_2_–CH_2_NH_2_^+^CH_2_Ger (see Table 1)), which defined the relative orientation of 2-adamantyl and geranyl groups. The conformation around the (2-Ad)CH_2_CH_2_–NH_2_^+^CH_2_Ger dihedral was *anti*. In (B) are shown the conformations generated by rotation around the C_1,Ad_C_2,Ad_–NHCH_2_ dihedral (see Table 3) which defined the position of axial NHCH_2_ as regards the cyclohexane subunit of 2-adamantyl group (geranyl group is shown with a light blue sphere); in the structure shown, in the right-hand part, the axial NHCH_2_ brings CH_2_ above the cyclohexane subunit of 2-adamantyl increasing steric repulsion (shown with two cross circular arc lines)

The rotation around the carbon–carbon bond, (2-Ad)NHCH_2_–CH_2_NH_2_^+^Ger, in SQ109 (**1a**), (Fig. 2A) generated the *gauche*(-), *gauche*( +), *anti* and *eclipsed* conformations and changed the relative orientation of the (2-Ad)NH and NH_2_^+^Ger groups and the hydrogen bonding profile of the ethylenediamine unit inside the MmpL3 receptor. The *gauche* conformations had the lowest free energy following the *anti* conformation, which was seriously destabilized, while the *eclipsed* conformer corresponds to the rotation barrier and was not stable. Both *gauche* conformations were stabilized since a hydrogen bond was formed between protonated and unprotonated nitrogen atoms in the ethylenediamine unit.

The rotation around (2-Ad)CH_2_CH_2_–NH_2_^+^CH_2_Ger dihedral favored the *anti* orientation (Table 2) since in the *gauche* conformation the steric energy increased due to repulsion between the geranyl and 2AdNH_2_^+^ groups. In these *gauche* conformations the geranyl group of the SQ109 (**1a**) could not adopt an extended structure that fits inside the narrow pore of the transporter.

The rotation around the C_1,Ad_C_2,Ad_–NHCH_2_ dihedral defined the position of the alkyl and the 2-adamantyl ammonium groups and the DFT calculations results are shown in Table 3. The C_1,Ad_C_2,Ad_–NHCH_2_ moiety can adopt only the equivalent two *gauche* conformations (which place the nitrogen of the ammonium group at C2-adamantyl position and C_n-1_ at symmetrical positions) due to the severe steric repulsions of the axial NHCH_2_ in the *anti* conformation with the cyclohexane subunit group of the 2-adamantyl group (Fig. 2B).

In summary, the DFT study showed that rotation of SQ109 around (2-Ad)NHCH_2_–CH_2_NH_2_^+^Ger dihedral favored two *gauche* conformations as minima while other conformers were unpopulated in both dielectric media as well as inside the transporter’s pore demonstrated by our MD simulations (see the next paragraph). Rotation around (2-Ad)NHCH_2_CH_2_–NH_2_^+^Ger bond favored an *anti* orientation and rotation around (2-Ad)–NHCH_2_CH_2_NH_2_^+^Ger bond showed that the position of NHCH_2_ group above the cyclohexane subunit was prohibited.

### MD simulations of the SQ109–MmpL3 complex

MmpL3 protein consists by 12 transmembrane (ΤΜ) α-helices and the TM region also contains two extra α-helices attached to the cytoplasmic membrane surface. We used the structure with PDB ID 6AJG [17] of the protein after excluding the C-terminus that has residues M1-E749. The TM domain consists by the following α-helices and their residues: TM1 (14–33), TM2 (174–199), TM3 (208–224), TM4 (238–264), TM5 (271–301), TM6 (306–338), TM7 (396–415), TM8 (552–576), TM9 (583–601), TM10 (625–648), TM11 (660–690), TM12 (697–728).

In the PDB ID 6AJG, [17] SQ109 (**1a**) binds to the transmembrane domain of the MmpL3 transporter, from G641 to F649. It disrupts the hydrogen bonding interactions between the two D-Y pairs, where proton translocation takes place, blocking activation of the transporter. In particular, the ethylenediamine group of SQ109 (**1a**) intervenes between the D256-Y646 and D645-Y257 pairs [14] and forms hydrogen bonds with Asp645. The X-ray structure (PDB ID 6AJG) [17] showed that the complex is stabilized through numerous van der Waals interactions between the geranyl-ethylenediamine moiety of SQ109 (**1a**) and surrounding amino acids of MmpL3, including I249, I253, I297 in the upper part and L642, Y257, Y646, L686 in the bottom part of the pocket while the 2-adamantyl group lies close to F260 and F649. Ethylenediamine molecule has p*K*_a,1_ = 10.71 and p*K*_a,2_ = 7.56 [43] and at pH 7.4 the mono and diprotonated species will be equally populated. However, since the basic amino group close to adamantyl group is more hindered to protonation, we assumed that the monoprotonated species must be predominated inside the hydrated MmpL3 pore, although actual protonation states are not known and would probably require neutron diffraction structures. Strikingly, in the X-ray structure (PDB ID 6AJG) [17] the ethylenediamine unit adopts a high energy conformation by rotation around its central carbon‒carbon bond, described in (2-Ad)NHCH_2_‒CH_2_NH_2_^+^Ger, having *eclipsed* the C-N bonds. Protein − ligand coordinates obtained from crystallography or cryo-em experiments provide a static model corresponding to a snapshot. While is not uncommon to find ligand conformers in complex with a protein that differ significantly from the lowest energy conformation in solution, due to stabilizing interactions inside the receptor, it is appropriate to judge the stability of the ligand’s conformation in the experimental structure using MD simulations, as previously suggested [44–48].

We performed 100 ns-MD simulations (two repeats, see Fig. S3) using the SQ109 (**1a**)-MmpL3 complex (PDB ID 6AJG) [17] embedded in a hydrated 1-palmitoyl-2-oleoyl-*sn*-glycero-3-phosphocholine (POPC) bilayer using the amber99sb force field (ff99sb) [49] and a 500 ns-MD simulation (Fig. S4) for testing the stability of the protein complex in longer time scale. For each simulation, initial atom velocities were assigned randomly and independently. Structure of SQ109 (**1a**)-MmpL3 complex with PDB ID 6AJG [17] has ~ 2.6 Å resolution which is higher than other structures resolved in refs [21, 22] while no other structure of MmpL3 in complex with an SQ109 (**1a**) analog has been resolved.

**Fig. 3:**
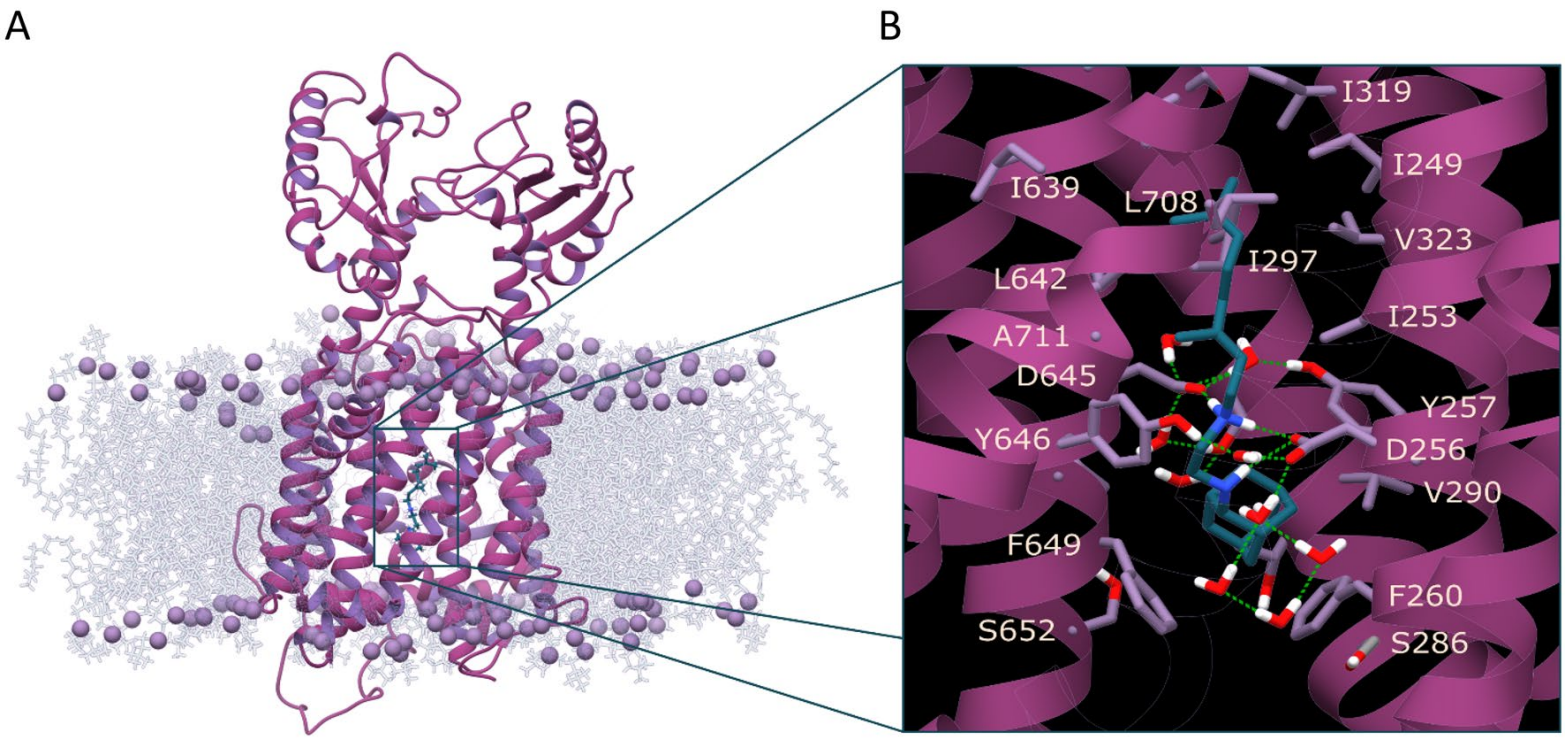
**A** Structure of MmpL3 in complex with SQ109 (**1a**) after 100 ns MD simulations with ff99sb [49]. **B** Close-up view of the binding site. SQ109 (**1a**) formed hydrogen bond interactions with the two D-Y pairs, D256-Y646 and D645-Y257. Color scheme for frames: Ligand = petrol sticks, receptor = purple ribbons, residues as light purple sticks. Hydrogen bonding interactions = green dashes. For the protein, the experimental structure of SQ109 (**1a**) in complex with MmpL3 (PDB ID 6AJG [17]) from *Ms*, was used as the starting structure for the MD simulations after excluding C-terminus, consisting by M1-E749 residues. The TM region included the following helices and their residues: TM1 (14–33), TM2 (174–199), TM3 (208–224), TM4 (238–264), TM5 (271–301), TM6 (306–338), TM7 (396–415), TM8 (552–576), TM9 (583–601), TM10 (625–648), TM11 (660–690), TM12 (697–728)

We observed a rotation of the carbon–carbon bond in the ethylenediamine unit that converts the *eclipsed* conformation of SQ109 (**1a**) in the MmpL3 X-ray structure (PDB ID 6AJG), [17] into the two possible *gauche* conformations, with no population of the *eclipsed* conformer over the whole trajectory. In each of the two *gauche* conformations the monoprotonated ethylenediamine unit formed stabilizing hydrogen bond interactions with both D256 and D645, as compared to the *eclipsed* conformation which can form hydrogen bonds only with D645. Thus, in the *gauche* conformation the NH_2_^+^ group of the monoprotonated ethylenediamine unit formed direct ionic hydrogen bonds with both D256 and D645 while the unprotonated NH acted as a donor in one direct and one water-mediated hydrogen bond with D256 (Fig. 3). We observed also that water molecules entered the pore forming hydrogen bonds between the monoprotonated ethylenediamine unit and side chains of Y257/Y646, D256/D645, S293 or carbonyl groups backbone peptide bonds. The geranyl-ethylenediamine moiety was tightly enclosed in a narrow area of the transporter’s pore with the geranyl chain surrounded by  amino acids Y257, I297, L642, Y646 while at the bottom wider part of the binding area the 2-adamantyl group had van der Waals contacts with F260 and F649.

### Simulations of the complexes of MmpL3 with SQ109 analogs 1b-i, 2

#### Docking calculations results

The X-ray structure of the MmpL3 − SQ109 (**1a**) complex (PDB ID 6AJG [17]) was used as the template structure for the docking calculations of ligands SQ109(**1a**), **1b-i, 2** with MmpL3 after excluding C-terminus, consisting of M1-E749 residues as previously described. The highest score docking pose of SQ109 (**1a**) inside the MmpL3 pore (ChemScore [50] scoring function) had a root-mean-square-deviations of heavy atoms (RMSD_ligand_) 1.7 Å compared to the structure with PDB ID 6AJG [17]. This suggested that the calculated docking poses could describe accurately the binding orientation of SQ109 analogues. The docking poses of the monoprotonated ethylenediamine SQ109 analogues **1b-i**, **2** showed that the new adamantyl moieties can fit in the empty region at the bottom of the binding area where for example the bigger alkyl adducts linked at C-2 adamantyl position can be accommodated. The docking calculations showed that addition of alkyl, aryl or heteroaryl adducts, e.g. Me, Et, nPr, nBu, Ph, Bn, Hex, 5-phenyl thiazol-2-yl (Ph-Thz) at the adamantyl C-2 of SQ109 (**1a**), in compounds **1b-i**, respectively, or replacement of the 2-adamantyl group by a 1-adamantyl-dimethylmethylene (C-1 dimethylmethylene) group in compound **2**, resulted in highest score docking poses having a *gauche*(−) or *gauche*( +) by rotation of (2-Ad)CH_2_–CH_2_NH_2_^+^CH_2_Ger that bind MmpL3 pore.

The docking algorithm produced 30 docking solutions. We visually inspected and realized that the first 4–5 highest docking solutions corresponded to a similar conformation of the ligand inside the receptor.

For the diprotonated SQ109 (**1a**) analogs, mainly the *gauche*(−) or *gauche*( +) by rotation of (2-Ad)CH_2_–CH_2_NH_2_^+^CH_2_Ger were obtained as highest score docking poses, but also in two cases the *anti* and *eclipsed* conformations were also observed (Table S2), since these later conformers stabilized inside the receptor with strong hydrogen bonding interactions despite their much lower stability in solution compared to the *gauche* conformation (Table 1). It was not unusual that the highest docking pose for each ligand was different since this reflected the high flexibility of the ligands and the random nature of the docking algorithm (genetic algorithm runs). For the MD simulations we used the highest scoring docking pose as starting conformation. We also tested as starting structure a conformation for the ligands **1b-i** resulted from superposition with the last snapshot from 100 ns-MD simulation of MmpL3-SQ109 (**1a**) complex.

### MD Simulations of SQ109 analogs in complex with MmpL3

Το explore the dynamic behavior between the ligands **1a-i**, **2** and MmpL3 we performed 100 ns-MD simulations (two repeats, see Fig. S3) with starting structure the docking pose with the highest score. Thus, these docking poses were embedded in hydrated POPC bilayers of ~ 140 lipids and subjected to MD simulations using ff99sb. For each simulation, initial atom velocities were assigned randomly and independently.We performed the 100 ns-MD simulations using as starting structure a conformation for the ligands 1b-i resulted from superposition with SQ109 (**1a**) but the results were similar.

**Fig. 4:**
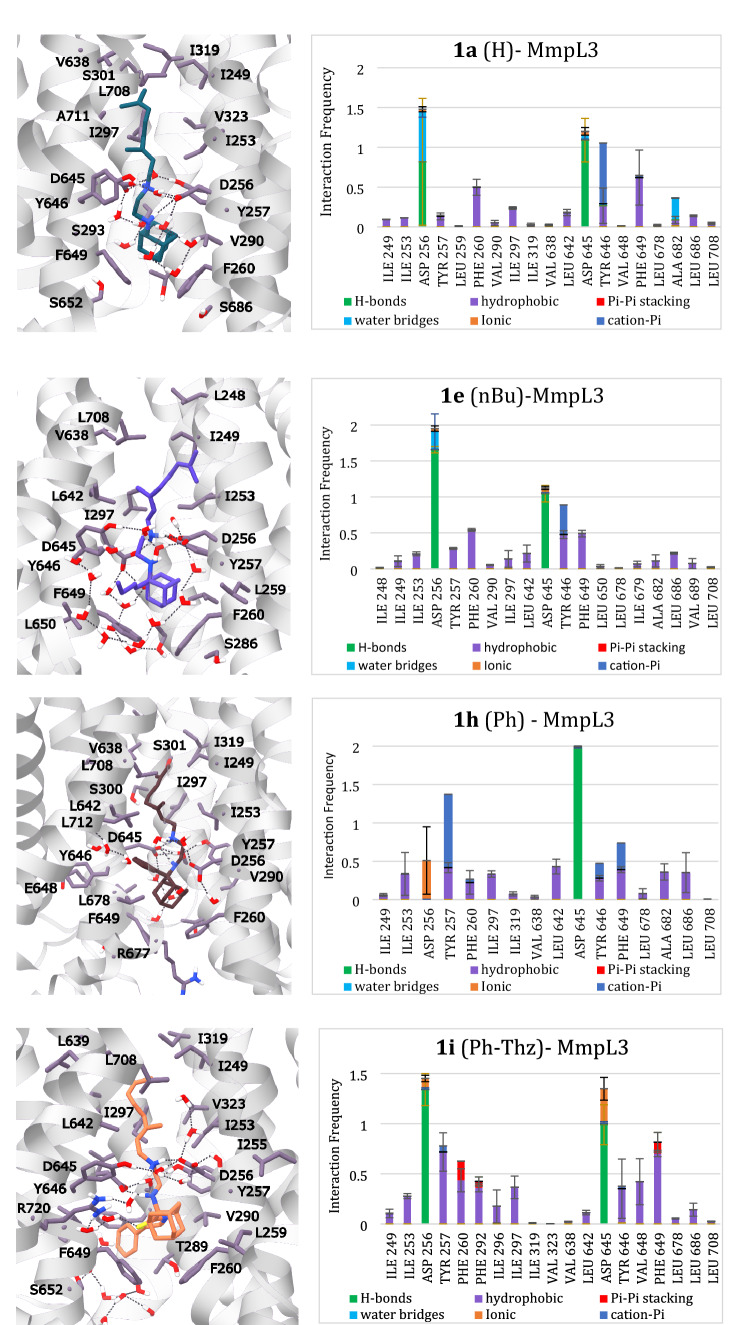
Last frames (left part) of monoprotonated ethylenediamine form for SQ109 (**1a**), and analogs **1e**, **h**, **i** inside the MmpL3 transporter in a POPC lipid bilayer from 100 ns-MD simulations with ff99sb. The receptor-ligand interaction frequency histograms plotted for the whole trajectory are shown on the right (the alkyl group connected at the adamantyl C-2 is shown inside the parenthesis following the compound number. Color scheme for frames: Ligand = petrol or purple or brown or orange sticks, receptor = white ribbons, residues in light purple sticks, hydrogen bonding interactions = dark grey dashes. For the protein, the experimental structure of SQ109 (**1a**) in complex with MmpL3 (PDB ID 6AJG 17) was used as the reference structure for the MD simulations after excluding C-terminus which included M1-E749 residues

For the sizeable alkyl substituents at C-2 adamantyl position,, e.g. in in analogs **1f**, **g**, **i**, 500 ns-MD simulations were also performed for testing the stability of the protein complexes in longer time scale (Fig. S4). For each simulation, initial atom velocities were assigned randomly and independently. We observed from the 100 ns-MD simulations or the 500 ns-MD simulations that the RMSD values of the protein TM Cα carbons converged after 10–40 ns with RMSD_protein_ (Ca TM) ≤ 2.1 Å, suggesting small changes compared to the X-ray structure (Fig. 4, S1, Table 4). A different stabilization between the full protein and the TM region was observed reflected by the different RMSD_protein_ (Ca full protein) and RMSD_protein_ (Ca TM) with values for the latter much smaller than the former. It is not unusual that the loops connecting the TM-helices are very flexible and increase the RMSD (Ca full protein). The ligands, which contained very flexible moieties such as the geranyl and ethylenediamine groups, shifted considerably from the starting docking pose, as revealed from the high values of RMSD ligand and in most cases the ligand binding conformation equilibrateds in a stable position inside the transporter’s pore after 70 ns (Fig. 4, S1, Table 4). The last frame described well the ligand–protein interaction frequency plots. Figure 4 shows last frames and ligand–protein interaction frequency plots for SQ109 (**1a**) and selected alkyl and aryl groups attached at the adamantyl C-2 of **1a** (R = H) in Fig. 1 including analogs **1b** (R = nBu), **1 h** (R = Ph), **1i** (R = Ph-Thz) while the last frames, ligand–protein interaction frequencies for the other analogs, as well as the RMSD plots from MD simulations can be found in Fig. S1.

The MD simulations showed that the monoprotonated ethylenediamine unit of the ligands **1b-i**, **2** adopted a *gauche* conformation, which favored hydrogen bonding interactions with 256 and/or D645 of MmpL3 (Fig. 4, S1). According to the MD simulations the complexes between **1b-i**, **2** and MmpL3 formed common van der Waals interactions with the protein’s residues along the narrow area of the transporter pore. Compared to SQ109 (**1a**) the geranyl-ethylenediamine moiety was surrounding similarly by the amino acid residues L642, Y646, Y257 while the 2-adamantyl group lied close to F260 and F649 at the bottom part of the binding area. However, the alkyl adducts increased the hydrophobic interactions at the bottom of the binding area with F260 and F649 and increased also the hydrophobic interactions with residues in the walls of narrow area of the pore, e.g. I253, see for example the analogs **1e**, **h**, **i** in Fig. 4. Water molecules formed hydrogen bonds between the monoprotonated ethylenediamine unit and receptor’s residues, as described previously for SQ109 (**1a**).

We explored the disposition of the important residues for binding of the ligands (D256, D645, Y257, Y646, F260, F649) along the MD simulation and checked for other frames that could accommodate better the ligands. The RMSD (Ca) of these important residues for binding were plotted (Fig. S5). The RMSD (Ca) converged to values that range between 0.5 and 1.5 confirming not important disposition of these residues along the MD simulation.

While the monoprotonated form of SQ109 (**1a**) and analogs most likely predominated inside the hydrated MmpL3 pore, we also performed MD simulations for the diprotonated species (Fig. S2). The MD simulations showed that the ligands in the diprotonated ethylenediamine form had similar coordinates inside the MmpL3 (Fig. S2) compared to the monoprotonated form.

### MM-GBSA binding free energy calculations

We applied the MM-GBSA method [29–31] using the OPLS2005 force field [51] for the calculation of binding free energies (Δ*G*_eff_) of ligands **1a-i, 2** inside the MmpL3 pore, using ensembles from 20 ns-MD simulations and calculated binding free energies without or with considering the membrane environment of the protein complex. For each simulation, initial atom velocities were assigned randomly and independently. Thus, we tested the membrane protein – ligand systems using an implicit membrane, i.e. a hydrophobic slab, [52–55] and considering explicitly water molecules inside the binding area [56]. The range of molecular weight of the ligands is 400–500 Da which corresponds to tripeptides [30] and their carbon skeleton was long enough to interact with many residues inside the receptor area. If a correct ranking of the binding affinities of **1b-i**, **2** for the MmpL3 pore could be accomplished with the MM-GBSA method this would be a significant reduction in computational resources, compared to the accurate binding free energy perturbation methods for **1b-i**, **2**—MmpL3 complexes in the POPC lipid bilayers containing ~ 10^5^ atoms.

However, the calculated mean values of three repeats for the monoprotonated forms (Table 4) and diprotonated forms (Table S2) showed that the MM-GBSA method (applied with or without modifications to consider the membrane environment of the protein complex using a hydrophobic slab [52–55] and the explicit waters inside the binding area) [56] did not afford valuable results. The binding free energy values were dispersed and did not follow any trend, see Table 4, S2. Indeed, as a calculation method, MM-GBSA can show large deviations (e.g. 4 kcal mol^−1^) in standard binding free energies compared to the experimental binding free energies. The method normally can provide useful results [57] for ranking of substituents [29] in the same series of ligands when the experimental binding affinities range is ~ 1000 or higher [30] which is not the case for **1b-i, 2** since their K_D_ values differ only by 4-fold.

Compared to the monoprotonated species, we observed that the diprotonated ethylenediamine moieties in compounds **1a-i**,**2** form stronger hydrogen bonding interactions with the receptor area. This agreed with the MM-GBSA binding free energy values for the monoprotonated forms compared to the diprotonated forms since in the latter case the values were significantly lower consistent with enhanced hydrogen bonding interactions between the ligands and the receptor’s binding site (Table 4, S2). Because of the stronger hydrogen bonding interactions, the ligands had smaller flexibility as was shown by the lower RMSD_protein_ (Ca TM) and lower RMSD_ligand_ (Fig. S1, S2, Table 4, S2).

**Table 4:** Ligand-MmpL3 binding free energies (Δ*G*_eff_) calculated using the MM-GBSA [29–31] method with the OPLS2005 [51] force field for the calculations of the intermolecular interactions without or with using a hydrophobic slab [51–55] to model the membrane environment of the protein, RMSD_ligand_ and RMSD_protein_ (Ca TMD) mean values from 100 ns-MD simulations for **1a-i, 2**

Cmp No	Structure	RMSD_ligand_^a^ (Å)	RMSD_protein_ (Ca)^b^ (Å)	RMSD_protein_ (Ca TMD) ^c^ (Å)	Δ*G*_eff_^d^ **(**kcal mol^−1^)
1a		3.29 ± 0.21	3.50 ± 0.12	1.17 ± 0.09	− 169.91 ± 7.37
1b		4.05 ± 0.28	4.28 ± 0.20	2.06 ± 0.07	− 131.80 ± 7.81
1c		3.02 ± 0.32	3.92 ± 0.19	1.83 ± 0.09	− 141.76 ± 9.72
1d		3.11 ± 0.21	3.57 ± 0.26	1.41 ± 0.08	− 159.92 ± 7.44
1e		4.00 ± 0.20	3.73 ± 0.17	1.29 ± 0.07	− 162.90 ± 8.18
1f		2.21 ± 0.21	3.61 ± 0.11	1.30 ± 0.05	− 160.48 ± 8.81
1 g		5.43 ± 0.28	3.33 ± 0.15	1.37 ± 0.06	− 143.97 ± 7.68
1 h		3.29 ± 0.18	2.71 ± 0.11	1.50 ± 0.07	− 153.60 ± 7.61
1i		3.12 ± 0.17	3.08 ± 0.17	1.38 ± 0.07	− 179.63 ± 8.75
2		3.21 ± 0.21	4.15 ± 0.30	1.26 ± 0.06	− 151.80 ± 8.74

The ligands have a monoprotonated ethylenediamine unit

^a^ Mean ± SD (Å); Ligand RMSD was calculated after superposition of each protein–ligand complex to that of the starting structure (snapshot 0) based on the C_α_ atoms of the protein, for the last 20 ns of the trajectories

^b^ Mean ± SD (Å); Protein RMSD was calculated for the C_α_ atoms of the whole protein, for the last 20 ns of the trajectories, using as starting structure snapshot 0 of the production MD simulation

^c^ Mean ± SD (Å); Protein RMSD was calculated for the C_α_ atoms of only the α-helices of the TM region, for the last 20 ns of the trajectories, using as starting structure snapshot 0 of the production MD simulation

^d^ Mean calculated effective binding free energy (kcal mol^−1^) between ligand and MmpL3 receptor from three repeats. Δ*G*_eff_ was calculated from the last 20 ns of the trajectories using 40 ps intervals (i.e. 500 frames per trajectory) using a MM-GBSA model that considered the membrane as hydrophobic slab. [51–55]

### Structure–activity relationships of SQ109 analogs against MmpL3 using alchemical binding free energy calculations with TI/MD

We previously measured [13] using SPR the binding affinities of SQ109 (**1a**) and the 9 active analogs **1b-i**, **2** against MtMmpL3 [23] with K_d_ values R = H (SQ109 (**1a**), 2060 μM); R = Me (**1b**, 248 μM); R = Et (**1b**, 190 μM); R = nPr (**1d**, 106 μM); R = nBu (**1e**, 108 μM); R = nHex (**1d**, 81 μM). It was observed that the Kd decreased showing tighter binding to MmpL3 as the R substituent at C-2 adamantyl (which is an H in SQ109) became larger and more hydrophobic. A similar effect was observed with phenyl (**1 h**, 136 μM); benzyl (**1 g**, 74 μM) (Table S1). The C-1 dimethylmethylene analog **2** had a K_d_ = 106 μΜ which was close to the isomeric **1d** (n-Pr) which had a K_d_ = 120 μM and **1i** (Ph-Thz) with a K_d_ = 91 μM.

The FEP/MD [58–60] or TI/MD [61, 33, 34] methods which are based on statistical mechanics can provide accurate results for relative binding free energies with an error 1 kcal mol^−1^ and have been applied in membrane protein–ligand complexes, e.g. GPCRs. [32, 35, 62–65]We applied the TI/MD method combined with a thermodynamic cycle method to examine if the binding profile of the ethylenediamine analogs **1b-i**, **2** was the same with SQ109 (**1a**) in its complex with MmpL3 (PDB ID 6AJG [17]). This might be likely if there is an agreement between calculated and experimental relative binding free energies for alchemical transformations between pairs of compounds **1a-i**, **2**. We performed TI/MD calculations for alchemical transformations in selected pairs of diamine SQ109 analogs that were not accompanied with large changes in ligand’s alkyl. Thus, we calculated perturbations by one or two methylene groups in the C-2 alkyl adduct (Table 5).

**Table 5:** Free energy perturbation values computed with the MBAR method [66] for alchemical simulations performed with TI/MD for pairs of ligands bound to Mmpl3

Alchemical transformation	ΔΔ*G*_b,exp_ (kcal mol^−1^)	ΔΔ*G*_b,TI/MD_ (kcal mol^−1^)	|ΔΔ*G*_b,TI/MD_—ΔΔ*G*_b,exp_| (kcal mol^−1^)
**1a** (H) → **1b** (Me)	− 1.30 ± 0.79	− 0.49 ± 0.06	0.81
**1b** (Me) → **1c** (Et)	− 0.16 ± 0.14	0.06 ± 0.08	0.23
**1c** (Et) → **1d** (n-Pr)	− 0.36 ± 0.29	− 0.87 ± 0.09	0.51
**1d** (n-Pr) → **1e** (n-Bu)	0.01 ± 0.30	0.20 ± 0.11	0.18
**1e** (n-Bu) → **1f** (n-Hex)	− 0.18 ± 1.01	− 1.42 ± 0.12	1.245
**1 h** (Ph) → **1 g** (Bn)	− 0.38 ± 0.02	− 1.83 ± 0.15	1.45

The end states in the alchemical calculations tested were similar to the structure in the corresponding complexes resulted from the converged 100 ns-MD simulations. This was checked to certify that the 2 ns-MD simulation in each λ-state was enough for the complexes to converge in an equilibrium structure. Two repeats of TI/MD calculations were performed for each alchemical transformation.

The effect in binding free energy by increasing the length of the alkyl chain by one methylene, which was examined with the alchemical perturbations **1a** (H) → **1b** (Me) or **1b** (Me) → **1c** (Et) or **1c** (Et) → **1d** (n-Pr) or **1d** (n-Pr) → **1e** (n-Bu), was to increase binding affinity (Table 5). As noted previously, in the 100 ns-MD simulations of MmpL3—**1a-e** complexes the RMSD_protein_ (Ca TMD) was ≤ 2.1 Å (Table 4). Thus, the last snapshots of the MD simulations were suitable starting structures for the TI/MD simulations of the studied perturbations (Fig. 4, S1).

The biggest change in experimental binding free energy was noted when  H (SQ109) changed to Me (**1b**), ΔΔ*G*_b,exp_ =− 1.30 kcal mol^−1^ ± 0.79 kcal mol^−1^, and when Ph (**1h**) chaged to Bn (**1 g**), ΔΔ*G*_b,exp_ = − 0.38 ± 0.02 kcal mol^−1^ or Et (**1c**) changed to Pr (**1d**), ΔΔ*G*_b,exp_ =− 0.36 kcal mol^−1^ ± 0.29 kcal mol^−1^.

The binding free energy changes for **1a** (H) → **1b** (Me) were ΔΔ*G*_b,exp_ =− 1.30 kcal mol^−1^ ± 0.79 kcal mol^−1^, ΔΔ*G*_b,TI/MD_ = − 0.49 ± 0.06 kcal mol^−1^, for **1b** (Me) → **1c** (Et) were ΔΔ*G*_b,exp_ = − 0.16 ± 0.14 kcal mol^−1^, ΔΔ*G*_b,TI/MD_ = 0.06 ± 0.08 kcal mol^−1^, for **1c** (Et) → **1d** (n-Pr) were ΔΔ*G*_b,exp_ = − 0.36 ± 0.29 kcal mol^−1^, ΔΔ*G*_b,TI/MD_ = − 0.87 ± 0.09 kcal mol^−1^ and for **1d** (n-Pr) → **1e** (n-Bu) were ΔΔ*G*_b,exp_ = 0.01 ± 0.30 kcal mol^−1^, ΔΔ*G*_b,TI/MD_ = 0.20 ± 0.11 kcal mol^−1^.

We considered next the perturbation in the C-2 adamantyl alkyl by two methylenes in **1e** (n-Bu) → **1f** (n-Hex) and we studied the change by one methylene from phenyl to benzyl in C-2 substituent in **1h** (Ph) → **1g** (Bn) where the perturbation in conformational space should be relatively important. The binding free energy changes were ΔΔ*G*_b,exp_ =—0.18 kcal mol^−1^ ± 0.01 kcal mol^−1^, ΔΔ*G*_b,TI/MD_ = -1.42 ± 0.15 kcal mol^−1^ and ΔΔ*G*_b,exp_ = -0.38 ± 0.02 kcal mol^−1^, ΔΔ*G*_b,TI/MD_ = -1.83 ± 0.15 kcal mol^−1^, respectively. We did not test larger perturbations that were not consistent with the method’s principles. [61]

In general, the deviation from experimental values was smaller than 1 kcal mol^−1^ when the perturbation was one methylene, e.g. for **1a** (H) → **1b** (Me), **1b** (Me) → **1c** (Et), **1c** (Et) → **1d** (n-Pr), **1d** (n-Pr) → **1e** (n-Bu), see Table 5. When the perturbation in the conformational space was bigger, e.g. was two methylene groups in **1e** (n-Bu) and **1f** (n-Hex) or from phenyl to benzyl in **1 h** (Ph) and **1 g** (Bn) the deviation was larger, i.e. 1.25 kcal mol^−1^ or 1.45 kcal mol^−1^, but in both these two cases was smaller than 1.5 kcal mol^−1^. Overall, the mean assigned error (mue) was 0.739 kcal mol^−1^ which was consistent with the fact that **1a-h** bind similarly with SQ109 (**1a**) to MmpL3 in its experimental structure (PDB ID 6AJG [17]), and that alkyl or aryl substituents at the adamantyl C-2 of SQ109 can fill the lipophilic region between Y257, Y646, F260 and F649 in MmpL3 pore and increasing the binding affinity.

## Discussion

SQ109 (**1a**) [5] is an ethylenediamine-based inhibitor of MmpL3 undergoing clinical trials [3, 4] that also has activity against a broad range of bacteria, protozoa and even some yeasts/fungi [26]. Previous research suggested that SQ109 (**1a**) can block the MmpL3-mediated transport of trehalose monomycolates [5, 6] through preventing the proton transportation by (a) binding directly to the transporter’s pore [17, 23] in *Mtb*, as supported by the X-ray structure of the MmpL3 from M. *smegmatis* in complex with SQ109 (**1a**) (PDB ID 6AJG [17]) or (b) indirectly [7, 26, 13, 67, 68] which in principle can be accomplished by membrane structure perturbation [7, 26, 32, 44, 56, 57] leading to increased membrane lipid disorder/fluidity and, arguably, to uncoupler activity on the PMF [5, 7, 24, 25].

The sequence of MmpL3 is highly conserved across *Mycobacteria* [68]. Of the MmpL proteins encoded by mycobacterial genomes, MmpL3 and MmpL11 are the only MmpL genes conserved across *Mycobacteria* [16]. The importance of MmpL3 is illustrated by the fact that it is the only MmpL gene that cannot be successfully knocked out in *Mtb* [15]. That MtMmpL3 could rescue the viability of the *Ms* ΜmpL3 knockout mutant further indicates that these ΜmpL3 orthologs have highly conserved functions [6]. Therefore, *Ms* MmpL3 is a reasonable model for the *Mtb* counterpart since the two MmpL3 orthologs can substitute each other to function [19].

Here, based on SPR data we previously obtained [13] (Table S1) showing the binding of **1a-i, 2** to MtMmpL3 and the tighter binding of the bigger adducts at C-2 adamantyl group, we investigated the binding profile of compounds **1b-i, 2** using MD simulations and alchemical relative binding free energy calculations based on the X-ray structure of the MmpL3-SQ109 (**1a**) complex from *Ms* (PDB ID 6AJG [17]).

We performed MD simulations of the X-ray structure with PDB ID 6AJG [17] in POPC bilayers containing ~ 140 lipids and showed that the *eclipsed* conformer observed in the X-ray structure represents the transition state for rotation around NHCH_2_-CH_2_NH_2_^+^ dihedral compared to the preferred *gauche* conformations inside the MmpL3 which we observed  in docking calculations and MD simulations.

To fully understand the conformational properties of SQ109 (**1a**) we performed DFT calculations of the *gauche* and *anti* conformations generated by rotation around (2-Ad)NHCH_2_–CH_2_NH_2_^+^Ger, (2-Ad)NHCH_2_CH_2_–NH_2_^+^CH_2_Ger and C_1,Ad_C_2,Ad_–NHCH_2_CH_2_NH_2_^+^Ger bonds. Thus, we calculated the free energies of SQ109 (**1a**) conformation in an implicit water environment (*ε* = 80) or in a lipophilic environment (chloroform, *ε* = 4.8) using the B3LYP-D3/6-31G(d,p) [69] theory (Tables 1–3, Fig. 2) and identified two *gauche* conformations as minima by rotation around AdNHCH_2_–CH_2_NH_2_^+^Ger dihedral. These *gauche* conformations were stabilized with a hydrogen bond between the protonated ammonium group and the unprotonated nitrogen; the next more stable was the *anti* conformation, being more than ~ 9 kcal mol^−1^ higher in energy and thus unpopulated (Table 1, Fig. 2), while the eclipsed conformer observed in the X-ray structure represents the transition state by rotation around AdNHCH_2_-CH_2_NH_2_^+^Ger bond. In both dielectric media and inside the transporter’s pore, rotation around AdNHCH_2_CH_2_–NH_2_^+^CH_2_Ger bond favored the *anti* orientation (Table 2), in agreement with the extended geranyl chain structure that fits inside the narrow pore of MmpL3 transporter, since in the *gauche* conformation the steric energy increased due to repulsion between the geranyl and NH_2_^+^(2-Ad) groups. The DFT calculations for the C_1,Ad_C_2,Ad_–NHCH_2_CH_2_NH_2_^+^Ger bond rotation, which defined the position of axial NHCH_2_ as regards the cyclohexane subunit of adamantyl group, showed that the position of axial NHCH_2_ brings CH_2_ above the cyclohexane subunit is prohibited as increasing considerably the steric repulsion.

In addition, we performed MD simulations of the complexes between MmpL3 and **1b-i, 2** which showed that, comparatively to SQ109 (**1a**) the ligands formed also hydrogen bonding interactions with D256 and/or D645 of MmpL3 (Fig. 4, S1) and had common van der Waals interactions with the protein’s residues along the transporter’s pore axis. In these complexes the geranyl-ethylenediamine moiety was surrounding by the amino acid residues L642, Y646, Y257 while the 2-adamantyl group lied close to F260 and F649 at the bottom part of the binding area. We observed that increasing the length of the alkyl chain the hydrophobic interactions with F260 and F649 were increased as well as with residues in the pore, e.g. I253 (Fig. 4, S1) which was consistent with the SPR binding affinities.

To further confirm that the new SQ109 analogs **1b-i, 2** bind to the Mmpl3 pore according to the experimental structure of SQ109(**1a**) bound to MmpL3 (PDB ID 6AJG [17]), we compared the calculated binding free energy values with the MM-GBSA method [29–31] (without or with using an implicit membrane model [52–55] and considering also explicitly water molecules inside the binding area [56]) with the binding strength ranking from the experimental SPR results. The results showed that no valuable correlation was observed.

We performed alchemical relative binding free energy calculations using the accurate TI/MD method [61] which, along with FEP/MD method, [58] have been shown to perform with an error of 1 kcal mol^−1^. For full accuracy, we included in the TI/MD calculations the whole protein-membrane system consisting of 10^5^ atoms. We applied the TI/MD method in alchemical transformations including changes in alkyl adduct at C-2 adamantyl by one or two methylene groups (Table 5) and examined how the calculated relative binding free energies were compared with experimental values that we measured using SPR (Table S1) [13]. For one methylene perturbations the deviation from experimental values was smaller than 1 kcal mol^−1^ and for two methylenes or for Ph to Bn perturbations the deviation was bigger, but smaller than 1.5 kcal mol^−1^. We observed a mue = 0.74 kcal mol^−1^ that is  consistent with the fact that alkyl or aryl substituents at the adamantyl C-2 of SQ109 (**1a**) can fill the empty lipophilic region close to F260 and F649.

Altogether, the MD simulations data that we produced based on the X-ray structure of the SQ109 (**1a**) and MmpL3 complex (PDB ID 6AJG [17]) agreed that our previously synthesized SQ109 (**1a**) analogs **1b-i, 2** bind to the same binding area with SQ109 (**1a**). Compared to SQ109 (**1a**), in the analogs with larger alkyl or aryl adducts in the adamantane ring, the geranyl-ethylenediamine moiety was similarly surrounding by the amino acid residues L642, Y646, Y257. However, the larger adducts at 2-adamantyl carbon can fit close to F260 and F649 increasing the hydrophobic interactions at the bottom of the binding area.

## Methods

### DFT calculations

For the DFT calculations was used the B3LYP functional [36–38] in combination with D3 Grimme’s correction for dispersion. [39, 69] All structures were fully optimized at B3LYP-D3/6-31G(d,p) level using the GAUSSIAN 03 [70] package; frequency calculations were also performed to locate minima.

### Ligands preparation

The 2D structures of the compounds SQ109(**1a**), **1b-i, 2** were sketched with Marvin Program (Marvin version 21.17.0, ChemAxon, https://www.chemaxon.com), model-built with Schrödinger 2017–1 platform (Schrödinger Release 2021–1**:** Protein Preparation Wizard; Epik, Schrödinger, LLC, New York, NY, 2021; Impact, Schrödinger, LLC, New York, NY; Prime, Schrödinger, LLC, New York, NY, 2021) and the compounds' 3D structures in their monoprotonated form were energy minimized using the conjugate gradient method, the MMFF94 [71] force field and a distance-dependent dielectric constant of 4.0 until a convergence threshold of 2.4 10^–5^ kcal mol^−1^ Å^−1^ was reached. The ionization state of the compounds at pH 7.5 were checked using the Epik program [72] implemented in Schrödinger suite (Prime Version 3.2, Schrödinger, LLC, New York, NY, 2015). Τhe most likely state for the ethylenediamine unit is the mono- protonated but we also performed all the calculations for the diprotonated state as well.

### Docking calculations

The X-ray structure of the MmpL3 − SQ109 (**1a**) complex (PDB ID 6AJG [17]) was used as the template structure for the docking calculations of ligands SQ109(**1a**), **1b-i, 2** with MmpL3. The part of the protein sequence that extended to the periplasmic area and included amino acids F750-H929, was deleted as this part was very distant from the binding site. Additionally, the 34 amino acid sequence Κ355-G388 that was missing from the X-ray structure (PDB ID 6AJG [17]) was added using the Prime module of Maestro (Schrödinger Release 2021–1**:** Protein Preparation Wizard; Epik, Schrödinger, LLC, New York, NY, 2021; Impact, Schrödinger, LLC, New York, NY; Prime, Schrödinger, LLC, New York, NY, 2021). In the next step, the MmpL3 − SQ109 (**1a**) complex was optimized using the Protein Preparation Wizard implementation in Schrödinger suite (Schrödinger Release 2021–1**:** Protein Preparation Wizard; Epik, Schrödinger, LLC, New York, NY, 2021; Impact, Schrödinger, LLC, New York, NY; Prime, Schrödinger, LLC, New York, NY, 2021). [73] In this process, the bond orders and disulfide bonds were assigned, and missing hydrogen atoms were added. The N- and C-termini of the protein model were capped by acetyl and N-methyl-amino groups, respectively. All hydrogens of each protein complex were minimized with the OPLS2005 force field [74, 75] by means of Maestro/Macromodel 9.6 [76] using a distance-dependent dielectric constant of 4.0. The molecular mechanics minimizations were performed with the conjugate gradient method and a threshold value of 2.4 10^–5^ kcal mol^−1^ Å^−1^ as the convergence criterion. Each protein was subjected in an all atom minimization using the OPLS2005 [74, 75] force field with heavy atom root mean square deviation (RMSD) value constrained to 0.30 Å until the RMS of conjugate-gradient reached values < 0.05 kcal·mol^−1^·Å^−1^. Then SQ109 (**1a**), utilized as a reference ligand, and the apo protein MmpL3, utilized as template protein, were saved separately or the docking calculations of the tested compounds SQ109(**1a**), **1b-i, 2** to MmpL3 using GOLD software [77] (GOLD Suite, Version 5.2; Cambridge Crystallographic Data Centre: Cambridge, U.K., 2015. GOLD Suite, version 5.2; Cambridge Crystallogr. Data Cent. Cambridge, U.K., 2015) and ChemScore [50] as the scoring function. Each compound was docked in the binding site of SQ109(**1a**) in area of 10 Å around the experimental coordinates of SQ109 (**1a**) and 30 genetic algorithm runs were applied for each docking calculation. The “allow early termination” option, which terminated ligand conformational sampling if the top three solutions had an RMSD difference less than 1.5 Å was inactivated, and the “Generate Diverse Solutions” option, which sets the smallest inter-cluster RMSD to 1.5 Å, was activated. All other parameters were used with their default values. We performed the docking calculations also for the SQ109 analogs **1a-i, 2** in the diprotonated form of ethylenediamine unit. The visualization of produced docking poses was performed using the program Chimera, [78] and the top-scoring docking poses were used as starting structures for the complexes for MD simulations to investigate the binding profile of the SQ109 (**1a**) and analogs **1b-i, 2** inside the MmpL3 pore.

### MD simulations

Each protein–ligand complex from docking calculations was inserted in a pre-equilibrated hydrated POPC membrane bilayer according to Orientations of Proteins in Membranes (OPM) database [79]. The protein was added in the hydrated lipid bilayer extended by 10 Å, 10 Å, 18 Å in *x*, *y*, *z* axes from the protein, consisting by ca. 140 lipids and 22,000 TIP3P water molecules, [80] using the System Builder utility of Desmond v4.9 (Schrödinger Release 2021–1**:** Desmond Molecular Dynamics System, D. E. Shaw Research, New York, NY, 2021. Maestro-Desmond Interoperability Tools, Schrödinger, New York, NY, 2021). Sodium and chloride ions were added randomly in the water phase to neutralize the systems and reach the experimental salt concentration of 0.150 M NaCl. The total number of atoms of the complex was approximately 100,000 and the orthorhombic simulation box dimensions was (86 × 83 × 141 Å^3^ ) and applied periodic boundary conditions. We used the Desmond Viparr tool to assign amber99sb [49] force field parameters for the calculations of the protein and lipids and intermolecular interactions, and Generalized Amber Force Field (GAFF) [81] for assigning parameters to ligands. Ligand atomic charges were computed according to the RESP procedure [82, 83] using the Gaussian03 program [70] and the antechamber module of Amber18 [84].

The MD simulation of each protein–ligand complex inside the lipid bilayer was performed using the default protocol provided with Desmond v4.9 program. Thus, the MD simulations protocol consisted of a series of MD simulations designed to relax the system, while not deviating substantially from the initial coordinates. During the first stage, a simulation was run for 200 ps at a temperature of 10 K in the NVT ensemble (constant number of atoms, volume and temperature), with solute-heavy atoms restrained by a force constant of 50 kcal mol^−1^ Å^−2^. The temperature was raised to 310 K during a 200 ps MD simulation in the NPT ensemble (constant number of atoms, pressure and temperature), with the same force constant applied to the solute atoms. The temperature of 310 K was used in MD simulations to ensure that the membrane state was above the main phase transition temperature of 296 K for POPC bilayers. [85] The heating was then followed by equilibration simulations. First, two 1 ns stages of NPT equilibration were performed. In the first 1 ns stage, the heavy atoms of the system were restrained by applying a force constant of 10 kcal mol^−1^ Å^−2^, and in the second 1 ns stage, the heavy atoms of the protein–ligand complex were restrained by applying a force constant of 2 kcal mol^−1^ Å^−2^ to equilibrate water and lipid molecules. In the production phase, the relaxed systems were simulated without restraints under NPT ensemble conditions for 100 ns or 500 ns.

Particle Mesh Ewald (PME) [86, 87] was employed to calculate long-range electrostatic interactions with a grid spacing of 0.8 Å. The SHAKE method was used to constrain heavy atom-hydrogen bonds at ideal lengths and angles [88]. Van der Waals and short-range electrostatic interactions were smoothly truncated at 10 Å. The Nosé-Hoover thermostat [89] was utilized to maintain a constant temperature in all simulations, and the Martyna-Tobias-Klein method [90] was used to control the pressure. The equations of motion were integrated using the multistep reversible reference system propagator algorithms (RESPA) integrator [91] with an inner time step of 2 fs for bonded interactions and non-bonded interactions within a cutoff of 10 Å. An outer time step of 6.0 fs was used for non-bonded interactions beyond the cutoff. Replicas of the system were saved every 10 ps. Within the 100 ns-MD simulation time, the total energy (not shown) and RMSD_protein_ (C_α_ TM) atoms reached a plateau, and the systems were considered equilibrated and suitable for statistical analysis (see Table 4, S2). The calculated RMSD_protein_ (C_α_ TM) for the last 50 ns was < 2.0 Å (see blue curves in Fig. S1). Two MD simulation repeats (Fig. S3) were performed for each system using the same starting structure and by applying in the MD simulations randomized velocities. We also used the same protocol and performed the MD simulations for the SQ109 analogs **1a-i, 2** in their doubly protonated form of ethylenediamine unit in complex with MmpL3 (Fig. S2). All the MD simulations with Desmond or Amber software were run on GTX 1060 GPUs in lab workstations or the ARIS Supercomputer.

The visualization of the trajectories was performed using the graphical user interface (GUI) of Maestro and the protein–ligand interaction analysis was done with the Simulation Interaction Diagram (SID) tool, available with Desmond v4.9 program. For hydrogen bonding interactions, a 2.5 Å distance between donor and acceptor heavy atoms, and an angle ≥ 120° between donor-hydrogen-acceptor atoms and ≥ 90° between hydrogen-acceptor-bonded atom were applied. Non-specific hydrophobic contacts were identified when the side chain of a hydrophobic residue fell within 3.6 Å from a ligand’s aromatic or aliphatic carbon, while π-π interactions were characterized by stacking of two aromatic groups face-to-face or face-to-edge. Water-mediated hydrogen bonding interactions were characterized by a 2.7 Å distance between donor and acceptor atoms, as well as an angle ≥ 110° between them.

### MM-GBSA calculations

For these calculations, structural ensembles were extracted in intervals of 40 ps from three 20 ns MD simulation repeats for each complex running with randomized velocities. Prior to the calculations all water molecules, ions, and lipids were removed, except 20 waters in the vicinity of the ligand, [92] and the structures were positioned such that the geometric center of each complex is located at the coordinate origin. The MD trajectories were processed with the Python library MDAnalysis [93] in order to extract the 20 water molecules closest to any atom in the ligand for each of the 501 frames. During the MM-PBSA calculations, the explicit water molecules were considered as being part of the protein. Binding free energies of compounds in complex with MmpL3 were estimated using the 1-trajectory MM-GBSA approach. [29–31] The binding free energy for each complex was calculated using Eqs. (1)-(6)

1 $${\Delta G}_{bind}= {\langle {G}_{complex}-{G}_{protein}-{G}_{ligand}\rangle }_{complex }$$

2 $${G}_{i}={{V}_{MM}-T\langle {S}_{MM}\rangle + {\Delta G}_{solv}}$$

3 $${V}_{MM}={V}_{bonded}+{V}_{coul}+{V}_{LJ}$$

4 $${\Delta G}_{solv}={\Delta G}_{P}+\Delta {G}_{NP}$$

The binding free energy for each complex can be calculated according to Eq. (5)

5 $${\Delta G}_{bind}= {\langle {\Delta {\rm E}}_{coul}+{\Delta E}_{LJ}\rangle -T\langle \Delta {S}_{MM}\rangle + {\Delta \Delta G}_{solv}}$$

and after neglecting entropy Eq. (5) is converted to Eq. (6)

6 $${\Delta G}_{eff}= {\langle {\Delta {\rm E}}_{coul}+{\Delta E}_{LJ}\rangle + {\Delta \Delta G}_{solv}}$$

In Eqs. (1)-(4) *G*_*i*_ is the free energy of system *i*, that being the ligand, the protein, or the complex; *V*_MM_ is the potential energy in vacuum as defined by the molecular mechanics (MM) model, which is composed of the bonded potential energy terms (*V*_bonded_) and nonbonded Coulombic (*V*_coul_) and Lennard–Jones (*V*_LJ_) terms; *S*_MM_ is the entropy; Δ*G*_solv_ is the free energy of solvation for transferring the ligand from water in the binding area calculated using the PBSA model, composed by a polar (Δ*G*_P_) and nonpolar (Δ*G*_NP_) term; *T* is the temperature and angle brackets represent an ensemble average. Molecular mechanics energies for Lennard–Jones (*V*_LJ_) and Coulombic elecrostatic (Coul) *V*_coul_ were calculated with OPLS2005 [94] force field; in these calculations Δ*V*_bonded_ = 0 as the single trajectory method was adopted and Δ*V*_MM_ = Δ*V*_LJ_ and Δ*V*_coul_. The polar part of the solvation free energy was determined by calculations using the Generalized-Born model. [95]The nonpolar term was considered proportional to the solvent accessible surface area (SASA), Δ*G*_NP_ = γ · SASA, where γ = 0.0227 kJ mol^−1^ Å^−2^. Because the SQ109 analogues tested are very similar entropy term was neglected and Δ*G*_bind_ is termed as effective binding energy, Δ*G*_eff_ which is calculated according to Eq. (6). [96] We applied a dielectric constant *ε*_solute_ = 1 to the binding area and to account for the lipophilic environment of the protein an heterogeneous dielectric implicit membrane model was used along the bilayer z-axis. [52–55]. The post-processing thermal_mmgbsa.py script of the Schrodinger Suite was used which takes snapshots from the MD simulations trajectory and calculated Δ*G*_eff_ according to Eq. (6).

### Alchemical TI/MD binding free energies calculated with MBAR method

#### Method’s principles

**Fig. 5:**
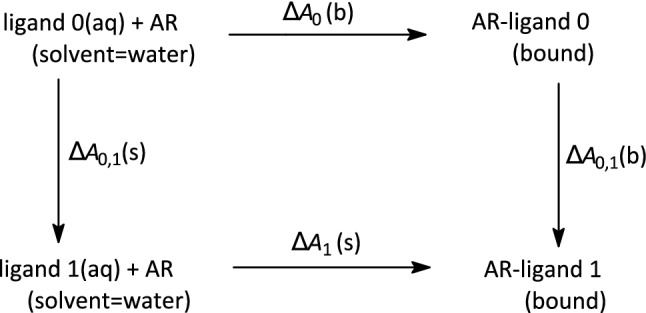
Thermodynamic cycle used for the calculation of relative binding free energies

The TI/MD method has been described [61]. Free energy is a state function, and thus the free energy difference between states is independent of the path that connects them. To compare two ligands 0 and 1 binding to a receptor the calculation of ${\Delta A}_{1}\left(b\right)$ and ${\Delta A}_{0}\left(b\right)$, respectively, is needed and then the difference ${\Delta \Delta A}_{0\to 1}$ (b) or ${\Delta \Delta A}_{0,1}$ (b). The calculation of ${\Delta A}_{1}\left(b\right)$ and ${\Delta A}_{0}\left(b\right)$ is computationally demanded and subjected to big errors because its includes large changes between the two states. Thus, the calculation of the relative binding free energies for two ligands bound to MmpL3 (for the 6 pairs of ligands shown in Table 5, respectively) was performed instead using the MBAR method [66] and applying a thermodynamic cycle. [33, 34, 97] (Fig. 5), ie. using the Δ*G* values obtained for the transformations of the ligands in the bound (b) and the solvent (s), i.e. water states, respectively, Δ*G*_0,1_(b) and Δ*G*_0,1_(s), according to Eq. (7)

7 $${\Delta \Delta A}_{b,0\to 1} or {\Delta \Delta A}_{b,\mathrm{0,1}}={\Delta A}_{1}\left(b\right)-{\Delta A}_{0}\left(b\right)={\Delta A}_{\mathrm{0,1}}\left(b\right)-{\Delta A}_{\mathrm{0,1}}\left(s\right)$$

Using this method, we calculated the difference between ${\Delta A}_{\mathrm{0,1}}\left(b\right) and {\Delta A}_{\mathrm{0,1}}\left(s\right)$ which corresponds to the unphysical alchemical transformation 0→1 in the bounds state and in the water state known as alchemical transformation which may be chosen to include small change (perturbation) of ligand structure, eg. H to CH_3_ at 2-position of adamantyl group, to lower the error for the free energy perturbation calculation ${\Delta A}_{\mathrm{0,1}}\left(b\right) or {\Delta A}_{\mathrm{0,1}}\left(s\right)$.

Because the phase space overlap between two states 0, 1 of interest can be near zero, doing free energy calculations for the two states alone will often have very large errors. Free energy is a state function, we can construct a thermodynamic path that takes us through a set of states that improves phase space overlap between states that can be unphysical. By this, we mean that intermediate states do not have to be observable experimentally. To put this mathematically, we can improve our results by constructing high phase space overlap intermediates and calculating our free energy difference ${\Delta \Delta A}_{0\to 1}$ by the sum of the binding free energy differences between the intermediate states.

Briefly, a thermodynamic parameter λ was used that smoothly connects states 0 and 1 through a λ-dependent potential *U*(*r*^*N*^; *λ*), such that *U*(*r*^*N*^; 0) = *U*_0_(*r*^N^) and U(*r*^*N*^; 1) = U_1_(*r*.^*N*^). The transformation was broken down into a series of *M* steps corresponding to a set of *λ* values *λ*_1_, *λ*_2_, …, *λ*_M_ ranging from 0 to 1, such that there was sufficient phase space overlap between neighboring intermediate *λ* states. TI computes the free energy change of transformation 0 → 1 by integrating the Boltzmann averaged d*U*(*λ*)/d*λ* as is described in Eq. (8)

8 $$\begin{array}{c}{\Delta {\rm A}}_{0\to 1}= {{\int }_{0}^{1}d\lambda {\langle \frac{dU\left({r}^{N};\lambda \right)}{d\lambda }\rangle }_{\lambda }}\\ =\hspace{0.17em}{\Delta {\rm A}}_{0\to 1}\approx {\sum }_{k=1}^{\rm M}{{w}_{k}\langle \frac{dU\left({r}^{N};\lambda \right)}{d\lambda }\rangle }_{{\lambda }_{k}}\end{array}$$

where the second sum indicates numerical integration over *M* quadrature points (*λ*_*k*_, for *k* = 1, …, *M*) with associated weights *w*_*k*_. A linear extrapolation between states can be applied for the construction of U_1_(*r*^*N*^; *λ*) while with Amber18 softcore potentials [34, 59, 98] the LJ and Coulomb term potentials are described according to Eq. (9)

9 $$\begin{array}{c}U\left({r}^{N};\lambda \right)={U}_{0}^{SC}\left({r}^{N};\lambda \right)+\lambda \Delta {U}^{SC}\left({r}^{N};\lambda \right)\\ ={U}_{0}^{SC}\left({r}^{N};\lambda \right)+\lambda \left({U}_{1}^{SC}\left({r}^{N};1-\lambda \right)-{U}_{0}^{SC}\left({r}^{N};\lambda \right)\right)\end{array}$$

MBAR [66] calculated the free energy difference between neighboring intermediate states using Eq. (10)

10 $${\Delta {\rm A}}_{\lambda \to \lambda +1}= {-lnln \frac{{\langle wexp\left(-\beta {U}_{\lambda +1}\right)\rangle }_{\lambda }}{{\langle wexp\left(-\beta {U}_{\lambda +1}\right)\rangle }_{\lambda +1}} }$$

where w is a function of *Α*(*λ*) and Α(*λ* + 1). The equation was solved iteratively to give the free energy change of neighboring states Δ*Α*(*λ* → *λ* + 1), which via combination yielded the overall free energy change. MBAR method has been shown to minimize the variance in the calculated free energies, by making more efficient use of the simulation data [66, 99–101].

#### TI/MD simulations protocol

For the TI/MD calculations performed with ff14sb, [102] the relaxed complexes of compounds **1a-e, g, h** with MmpL3 from the 100 ns-MD simulations in a POPC lipid bilayer with the ff99sb [49] were used as starting structures for the alchemical calculations. The setup procedure was the same as previously reported for the MD simulations with Amber18 program. [84] TI/MD calculations were also performed for the ligands in solution. The bond constraint SHAKE [88] algorithm was disabled for TI mutations in AMBER GPU-TI module pmemdGTI, [103] and therefore a time step of 1 fs was used for all MD simulations.

Initial geometries were minimized using 20,000 steps of steepest descent minimization at λ = 0*.*5. These minimized geometries were then used for simulations at all *λ* values. Eleven *λ* values were used, equally spaced between 0*.*0 and 1.0. Each simulation was heated to 310 K over 500 ps using the Langevin thermostat, [104, 105] with a collision frequency set to 2 ps^*−*1^. The Berendsen barostat [106] was used to adjust the density over 500 ps at constant pressure (NPT), with a target pressure of 1 bar and a 2 ps coupling time. Then, 500 ps of constant volume equilibration (NVT) was followed by 2 ns NVT production simulations. Energies were recorded every 1 ps, and coordinates were saved every 10 ps. Production simulations recalculated the potential energy at each *λ* value every 1 ps for later analysis with MBAR [66].

For each calculation, the 1-step protocol was performed, ie. disappearing one ligand and appearing the other ligand simultaneously, and the electrostatic and Van der Waals interactions were scaled simultaneously using softcore potentials from real atoms that were transformed into dummy atoms. [34] We carried out the calculations using the 1-step protocol which changed charges and Van der Waals interactions in a single simulation by activating both Lennard–Jones and Coulomb softcore potentials simultaneously, reducing the computational cost. [107] The 1-step protocol offers a less computational expensive and more accurate approach to free energy estimates according to recent studies. [98]

The final states 0 and 1 of the alchemical calculations 0 → 1 or 1 → 0, ie. the structures of ligand 0-AR and 1-AR complexes as resulted from the alchemical transformations were compared with these complexes structure resulted from converged 100 ns-MD simulations. This was performed to certify that the 2 ns MD simulation for each λ-state during the alchemical calculations was enough for the complexes 0-AR and 1-AR to converge to same structure with 100 ns-MD simulations. Two repeats were performed for the TI/MD calculation for each alchemical transformation (Table 5).

In summary, we performed for the ~ 100,000 atoms protein complexes studied here for the single protonation state 100 ns-MD simulations in 2 repeats × 9 ligands (18 MD simulations) and for the double protonation state 80 ns-MD simulations in 9 ligands (9 MD simulations). Additionally, we performed for 4 representative ligands 500 ns-MD simulations (4.52 μs MD simulation time). We performed the simulations using Desmond program, which performed much faster than Amber or Gromacs programs using an amber force field (ff99sb) that can fairly describe the protein conformation.

We tested the MM-GBSA calculations using ensembles from 20 ns-MD simulations with ff99sb for 10 ligands in 3 repeats × 2 protonation states (60 MD simulations). For the monoprotonated form of the ligands we tested an environment without or with an implicit model for membrane (2 environments). The simulation time for these simulations was 2.4 μs. We calculated the MM-GBSA interaction energies with free available OPLS2005 force field with Desmond software.

We used the last snapshot of the converged MD simulations and performed TI/MD simulations to calculate the relative binding free energies using alchemical perturbations and the amber software and ff14sb. Thus, we performed 2 repeats × 6 ligands × 10-λ values (120 2 ns-MD simulations). The total simulation time for the simulations performed in this study was 7.16 μs.

## Supplementary Information

Below is the link to the electronic supplementary material.

Supplementary file1 (DOCX 28603 KB)
Plots and frames from MD simulations of SQ109 analogs in monoprotonated and diprotonated form of ethylenediamine and SPR curves (Fig. S1-S5). Binding affinities (Table S1) from SPR against MtMmpL3 and biological activities against *Ms* and *Mtb* HN878 reproduced from ref. [13] and Table S2 with binding free energies (Δ*G*_eff_) calculated using MM-GBSA, mean values of RMSD_ligand_, RMSD_protein_ (Ca TMD) for diprotonated forms of ethylenediamines **1a-i, 2**.

## Abbreviations

cryo-EM: Cryo-electron microscopy
DFT: Density Functional Theory
GAFF: Generalized Amber Force Field
MD: Molecular dynamics
TB: Tuberculosis
MM-GBSA: Molecular mechanics generalized born surface area
Mtb: *Mycobacterium tuberculosis*
Ms: Mycobacterium smegmatis
MtHN878: *M. tuberculosis* HN878
OPM: Orientations of Proteins in Membranes
POPC: 1-Palmitoyl-2-oleoyl-sn-glycero-3-phosphocholine
RMSD: Root mean square deviation
TI/MD: Thermodynamic Integration coupled with MD simulations
TMM: Trehalose monomycolate
PMF: Proton motive force
RMSD: Root-mean-square deviation
SID: Simulation interaction diagram
