# Supplementary material for: Study of SQ109 analogs binding to mycobacterium MmpL3 transporter using MD simulations and alchemical relative binding free energy calculations
Source: J Comput Aided Mol Des. 2023 May 2;37(5-6):245–64. doi: 10.1007/s10822-023-00504-6 (PMC10232594; doi:10.1007/s10822-023-00504-6)
Supplement: Supplementary file 1 — Supplementary file1 (DOCX 28603 KB) Plots and frames from MD simulations of SQ109 analogs in monoprotonated and diprotonated form of ethylenediamine and SPR curves (Fig. S1-S5). Binding affinities (Table S1) from SPR against MtMmpL3 and biological activities against Ms and Mtb HN878 reproduced from ref. [13] and Table S2 with binding free energies (ΔGeff) calculated using MM-GBSA, mean values of RMSDligand, RMSDprotein (Ca TMD) for diprotonated forms of ethylenediamines 1a-i, 2. [file 10822_2023_504_MOESM1_ESM.docx]

SUPPORTING INFORMATION

**Study of SQ109 Analogs Binding to Mycobacterium MmpL3 Transporter Using MD simulations and Alchemical Relative Binding Free Energy Calculations**

Marianna Stampolaki, ^1^ Ioannis Stylianakis, ^1^ Helen I. Zgurskaya, ^2^ Antonios Kolocouris ^1,*^

^1^ Laboratory of Medicinal Chemistry, Section of Pharmaceutical Chemistry, Department of Pharmacy, National and Kapodistrian University of Athens, Panepistimiopolis-Zografou, Athens 15771, Greece

^2^ University of Oklahoma, Department of Chemistry and Biochemistry, Stephenson Life Sciences Research Center, 101 Stephenson Parkway, Norman, OK 73019-5251, USA

**Table of Contents**

| **Table S1**. Binding affinities from SPR against MtMmpL3 and biological activities against *Ms* and *Mtb* HN878. | S3 |
| --- | --- |
| **Table S2**. RMSD_lig_ mean values and RMSD_prot_ mean values for the protein (excluding C-terminus) or only transmembrane helices from 80ns-MD simulations for diprotonated ethylenediamine **1a-i, 2.** | S3-S4 |
| **Figure S1**. MD trajectories and protein-ligand interaction histograms for monoprotonated species **1a-i, 2**. | S4-S14 |
| **Figure S2**. MD trajectories and protein-ligand interaction histograms for diprotonated species **1a-h, 2**.  **Figure S3**. MD trajectories and protein-ligand interaction histograms of two repeats for monoprotonated species **1a-i, 2**.  **Figure S4**. MD trajectories and protein-ligand interaction histograms for monoprotonated species **1a-i, 2** from 500ns MD simulations.  **Figure S5**. MD simulations plots for residues that interact with ligand amine groups from 500ns MD simulations. | S15-S23  S23-S37  S37-S40  S40-S41 |
| Supporting Information References | S42 |

**Table S1**. Binding affinities from SPR against MtMmpL3 and biological activities against *Ms* and *Mtb* HN878 using the 2-states model taken from ref. ^1^

| **Comp. No** | **Chemical Structure** | **K_d_ (µM)** ^34^ | **IC_50_ (µM)** ^34^  *Ms* | **IC_50_ (µM)** ^1^  *Mtb* HN878 |
| --- | --- | --- | --- | --- |
| **1a** |  | 2060 ± 34.5 | 2.4 | 0.4 |
| **1b** |  | 248 ± 9.52 | 4.4 | 0.8 |
| **1c** |  | 190 ± 7.57 | 4.0 | 1.6 |
| **2** |  | 120 ± 15 | 15 | 3 |
| **1d** |  | 106 ± 12.2 | 4.3 | 3 |
| **1e** |  | 108 ± 12.2 | 2.5 | 3 |
| **1f** |  | 81 ± 38.9 | 4.7 | 3 |
| **1g** |  | 74 ± 30.3 | 6.0 | 1.6 |
| **1h** |  | 136 ± 29.5 | 5.8 | 1.6 |
| **1i** |  | 91 ± 31.1 | 21 | ND ^a^ |

*^a^* ND, not determined; *^b^* The Kd values are determined by global fitting of sensorgrams collected at different concentrations of an analyte to the same set of parameters. Residuals are the measure of goodness of the fit.

**Table S2**. Ligand-MmpL3 binding free energies (Δ*G*_eff_) calculated using MM-GBSA ^2,3^ method with OPLS2005 ^4^ force field for the calculations of the intermolecular interactions without or with using a hydrophobic slab ^5,6^ to model the membrane environment of the protein. RMSD_lig_ mean values and RMSD_prot_ mean values for the whole protein or only transmembrane helices from 80ns-MD simulations for **1a-h, 2.** The ligands have a diprotonated ethylenediamine unit.

| **Cmp No** | RMSD_lig_ ^a^ (Å) | RMSD_prot_ ^b^  (Å) | Δ*G*_eff_ ^c^  **(**kcal mol^−1^) | Δ*G*_eff_ ^d^  **(**kcal mol^−1^) | Conformation of the ethylenediamine unit of the ligand in the starting conformation ^e^ | Conformation of the ethylenediamine unit of the ligand in the last snapshot in the MD simulation |
| --- | --- | --- | --- | --- | --- | --- |
| **1a** | 1.72 ± 0.12 | 2.96 ± 0.12 | -192.19 ± 8.27 | -78.74 ± 4.61 | g(-) | g(-),g(+),g(+) |
| **1b** | 1.18 ± 0.11 | 3.40 ± 0.35 | -174.66 ± 6.46 | -73.06 ± 3.95 | g(-) | g(-),g(-),g(-) |
| **1c** | 2.12 ± 0.19 | 3.11 ± 0.10 | -170.74 ± 8.57 | -68.30 ± 5.54 | g(+) | g(+)g(-),g(+),g(+) |
| **1d** | 1.93 ± 0.14 | 2.75 ± 0.13 | -185.80 ± 7.25 | -93.05 ± 4.62 | anti | g(+),g(+),g(+) |
| **1e** | 2.09 ± 0.09 | 2.60 ± 0.10 | -195.04 ± 8.55 | -84.65 ± 4.73 | anti | g(-),g(-),g(-) |
| **1f** | 2.15 ± 0.25 | 4.13 ± 0.20 | -190.04 ± 9.02 | -80.18 ± 5.69 | eclipsed | g(+),g(+),g(+) |
| **1g** | 2.13 ± 0.10 | 2.70 ± 0.13 | -190.79 ± 11.7 | -85.88 ± 7.03 | g(+) | g(+),g(-),g(-) |
| **1h** | 2.21 ± 0.12 | 3.84 ± 0.20 | -173.45 ± 7.48 | -81.60 ± 4.55 | g(+) | g(+),g(-),g(-) |
| **2** | 1.37 ± 0.15 | 3.31 ± 0.12 | -184.31 ± 7.83 | -80.43 ± 7.21 | g(-) | g(-),g(-),g(+) |

^a^ Mean±SD (Å); Ligand RMSD is calculated after superposition of each protein-ligand complex to that of the starting structure (snapshot 0) based on the C_α_ atoms of the protein, for the last 20 ns of the trajectories.

^b^ Mean±SD (Å); Protein RMSD is calculated for the C_α_ atoms of the α-helices, for the last 20 ns of the trajectories, using as starting structure snapshot 0 of the production MD simulation.

^c^ Mean calculated effective binding free energy (kcal mol^-1^) between ligand and MmpL3 receptor from three repeats. Δ*G*_eff_ is calculated from the last 20 ns of the trajectories using 40 ps intervals (i.e. 500 frames per trajectory) using a MM-GBSA model that is taking into account the membrane as hydrophobic slab. ^5–7^

^d^ Mean calculated effective binding free energy (kcal mol^-1^) between ligand and MmpL3 receptor from three repeats. Δ*G*_eff_ is calculated from the last 20 ns of the trajectories using 40 ps intervals (i.e. 500 frames per trajectory) without taking into account the membrane. Using the last frame of the MD simulation trajectory, two additional 20 ns MD simulations were performed and structural ensembles were similarly selected.

^e^ Highest scored docking pose.

**Molecular dynamics results for complexes of monoprotonated SQ109 analogs with MmpL3**

**Figure S1.** Molecular dynamics results for complexes of monoprotonated SQ109 analogs with MmpL3. In panels **(A)** for **1a-i**, **2** are shown the chemical structures of the ligands and the RMSD plots for the Ca carbons (blue) for the full protein (upper RMSD plots) and the ligand heavy atoms (orange) or **(B)** only the RMSD plots for the Ca carbons (blue) of transmembrane helices and the ligand heavy atoms (orange). **(C)** Last frames of monoprotonated SQ109 analogs **1b** (Me), **2**, **1d** (Pr), **1f** (Hex), **1g** (Bn), **1c** (Et) inside the MmpL3 pore from 100ns-MD simulations with amberff99sb; ^8,9^. **(D)** Receptor-ligand interaction frequency histograms; (C), (D) for SQ109 or **1a** (H), **1e** (Bu),  **1h** (Ph), **1i** (Thz-Ph) are shown in the main manuscript. Color scheme for frames: Ligand=petrol or purple or orange or green or blue sticks, receptor = white ribbons, residues in light purple sticks, hydrogen bonding interactions=dark grey dashes. For the protein, the experimental structure of SQ109 in complex with MmpL3 (PDB ID 6AJG ^10^) was used. The protein structure (excluding C-terminus) consists of M1-E749 residues. The transmembrane domain consists of the following helices and their residues: TM1: 14-33, TM2: 174-199, TM3: 208-224, TM4: 238-264, TM5: 271-301, TM6:306-338, TM7:396-415, TM8: 552-576, TM9: 583-601, TM10: 625-648, TM11: 660-690, TM12: 697-728.

**Figure S1 (contd.).** MD simulation results for MmpL3 with SQ109 **(1a)**.

A

B

C


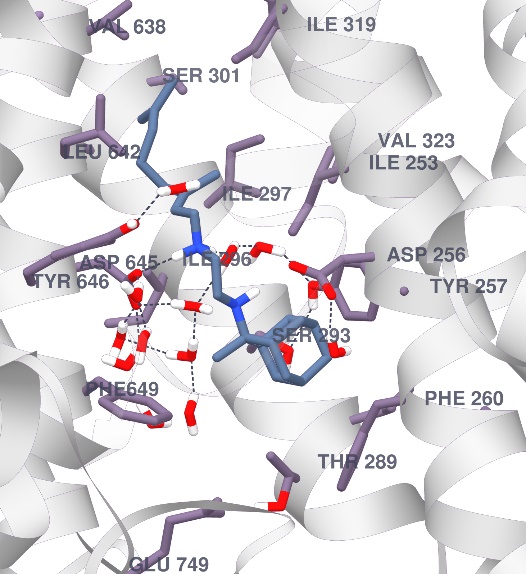


D

**Figure S1.** MD simulation results for MmpL3 with **1b**.

B

C


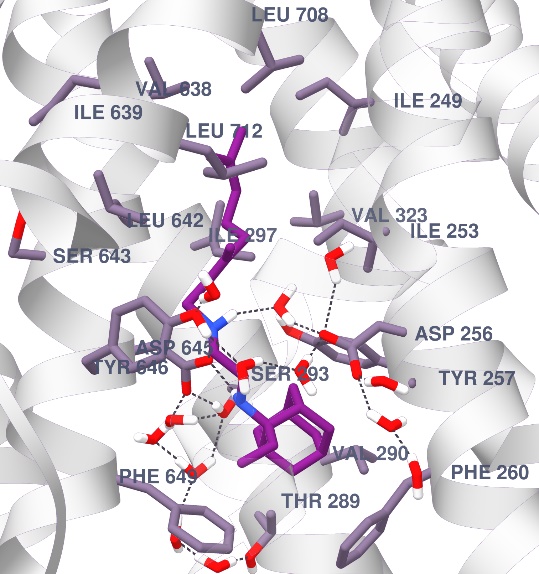


**Figure S1 (contd.).** MD simulation results for MmpL3 with **1c**.

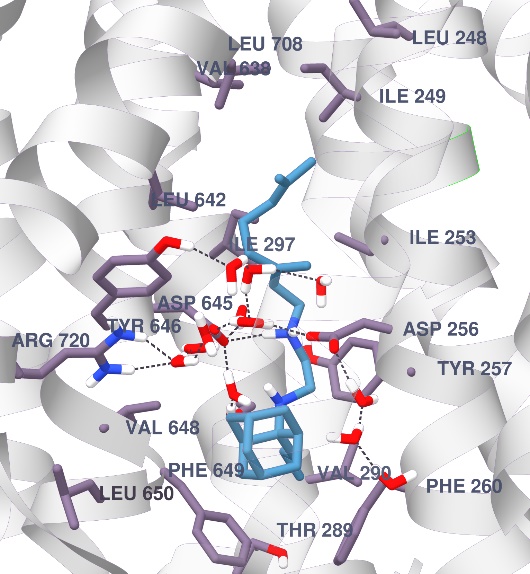


C

**Figure S1 (contd.).** MD simulation results for MmpL3 with **1d**.

**Figure S1 (contd.).** MD simulation results for MmpL3 with **1e**.

C


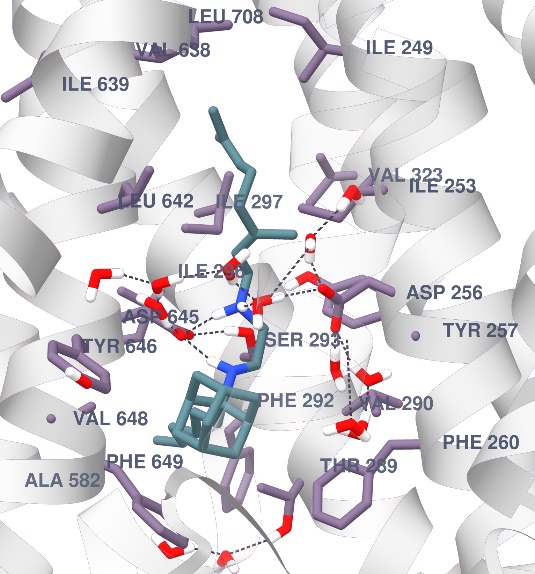


**Figure S1 (contd.).** MD simulation results for MmpL3 with **1f**.

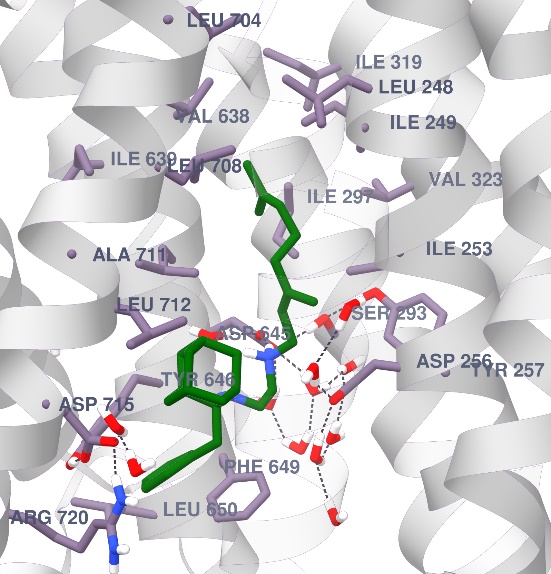


C

**Figure S1 (contd.).** MD simulation results for MmpL3 with **1g**.

**Figure S1 (contd.).** MD simulation results for MmpL3 with **1h**.

**Figure S1 (contd.).** MD simulation results for MmpL3 with **1i**.

A

B


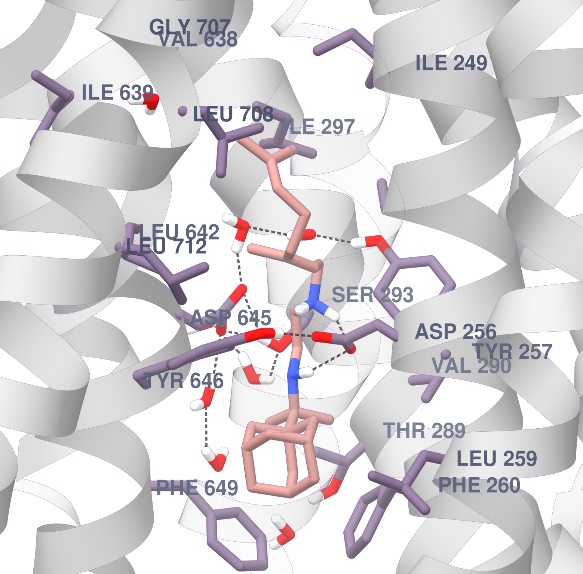


C

**Figure S1 (contd.).** MD simulation results for MmpL3 with **2**.

**MD simulations plots for the doubly protonated ethylenediamine forms of SQ109 analogs with MmpL3**

**Figure S2**. In panels (**A**) for **1a-1h** are shown the chemical structures of the ligands and the receptor-ligand interaction frequency histograms recorded from the 80 ns MD simulation trajectories of SQ109 analogues (doubly protonated ethylenediamine form) in complex with MmpL3. Hydrogen bonding interaction bar are depicted in green, Van der Waals in yellow, cation-pi in blue, pi-pi stacking in pink, water bridges in light blue and ionic in light green. (**B**) The RMSD plots for the Ca carbons (blue) of the full protein and the ligand heavy atoms (orange). For the protein, the experimental structure of SQ109 in complex with MmpL3 (PDB ID 6AJG) was used. The protein structure (excluding C-terminus) consists of M1-E749 residues. The transmembrane domain consists of the following helices and their residues: TM1: 14-33, TM2: 174-199, TM3: 208-224, TM4: 238-264, TM5: 271-301, TM6:306-338, TM7:396-415, TM8: 552-576, TM9: 583-601, TM10: 625-648, TM11: 660-690, TM12: 697-728.

**Figure S2.** MD simulation results for MmpL3 with **1a**.

**Figure S2 (contd.).** MD simulation results for MmpL3 with **1b**.

**Figure S2 (contd.).** MD simulation results for MmpL3 with **1c**.

**Figure S2 (contd.).** MD simulation results for MmpL3 with **1d**.

**Figure S2 (contd.).** MD simulation results for MmpL3 with **1e**.

**Figure S2 (contd.).** MD simulation results for MmpL3 with **1f**.

**Figure S2 (contd.).** MD simulation results for MmpL3 with **1g**.

**Figure S2 (contd.).** MD simulation results for MmpL3 with **1h**.

**Figure S2 (contd.).** MD simulation results for MmpL3 with **2**.

**100ns MD simulations plots of two replicas for the monoprotonated ethylenediamine forms of SQ109 analogs with MmpL3**

**Figure S3.** Molecular dynamics results of two replicas for complexes of monoprotonated SQ109 analogs with MmpL3. In panels **(A)** for **1a-i**, **2** are shown the chemical structures of the ligands and the receptor-ligand interaction frequency histograms as an average of two replicas: hydrogen bonding interaction bar are depicted in green, Van der Waals in yellow, cation-pi in blue, pi-pi stacking in red, water bridges in light blue and ionic in light green, **(B)** the RMSD plots for the Ca carbons (blue) of transmembrane helices and the ligand heavy atoms (orange) separately for the two replicas and as an average. For the protein, the experimental structure of SQ109 in complex with MmpL3 (PDB ID 6AJG) was used as the starting structure for the MD simulations after excluding C-terminus, consisting by M1-E749 residues. The transmembrane region included the following helices and their residues: TM1 (14-33), TM2 (174-199), TM3 (208-224), TM4 (238-264), TM5 (271-301), TM6 (306-338), TM7 (396-415), TM8 (552-576), TM9 (583-601), TM10 (625-648), TM11 (660-690), TM12 (697-728).

A

**Figure S3.** MD simulation results of two replicas for MmpL3 with **1a**.

**Figure S3 (contd.).** MD simulation results of two replicas for MmpL3 with **1b**.

**Figure S3 (contd.).** MD simulation results of two replicas for MmpL3 with **1c**.

**Figure S3 (contd.).** MD simulation results of two replicas for MmpL3 with **1d**.

**Figure S3 (contd.).** MD simulation results of two replicas for MmpL3 with **1e**.

**Figure S3 (contd.).** MD simulation results of two replicas for MmpL3 with **1f**.

**Figure S3 (contd.).** MD simulation results of two replicas for MmpL3 with **1g**.

**Figure S3 (contd.).** MD simulation results of two replicas for MmpL3 with **1h**.

**Figure S3 (contd.).** MD simulation results of two replicas for MmpL3 with **1i**.

**Figure S3 (contd.).** MD simulation results of two replicas for MmpL3 with **2**.

**500ns MD simulations plots for the monoprotonated ethylenediamine forms of SQ109 analogs with MmpL3**

**Figure S4**. In panels (**A**) for **1a-1i** are shown the chemical structures of the ligands and the receptor-ligand interaction frequency histograms recorded from the 500 ns MD simulation trajectories of SQ109 analogues (monoprotonated ethylenediamine form) in complex with MmpL3. Hydrogen bonding interaction bar are depicted in green, Van der Waals in yellow, cation-pi in blue, pi-pi stacking in red, water bridges in light blue and ionic in light green. (**B**) The RMSD plots for the Ca carbons (blue) of transmembrane helices and the ligand heavy atoms (orange). For the protein, the experimental structure of SQ109 in complex with MmpL3 (PDB ID 6AJG) was used as the starting structure for the MD simulations after excluding C-terminus, consisting by M1-E749 residues. The transmembrane region included the following helices and their residues: TM1 (14-33), TM2 (174-199), TM3 (208-224), TM4 (238-264), TM5 (271-301), TM6 (306-338), TM7 (396-415), TM8 (552-576), TM9 (583-601), TM10 (625-648), TM11 (660-690), TM12 (697-728).

**Figure S4.** MD simulation results for MmpL3 with **1a**.

**Figure S4 (contd.).** MD simulation results for MmpL3 with **1f**.

**Figure S4 (contd.).** MD simulation results for MmpL3 with **1g**.

**Figure S4 (contd.).** MD simulation results for MmpL3 with **1i**.

**500ns MD simulations plots for residues that interact with ligand amine groups**

**Figure S5**. The RMSD plots for the sum of Ca carbons of residues Asp256, Asp645, Tyr257, Tyr646, Phe260, Phe649. For the protein, the experimental structure of SQ109 in complex with MmpL3 (PDB ID 6AJG) was used as the starting structure for the MD simulations after excluding C-terminus, consisting by M1-E749 residues.

**Figure S5.** MD simulation results for MmpL3 with **1a**.

**Figure S5 (contd.).** MD simulation results for MmpL3 with **1f**.

**Figure S5 (contd.).** MD simulation results for MmpL3 with **1g**.

**Figure S5 (contd.).** MD simulation results for MmpL3 with **1i**.

**Supporting Information References**

1. Stampolaki, M. *et al.* Synthesis and Testing of Analogs of the Tuberculosis Drug Candidate SQ109 against Bacteria and Protozoa: Identification of Lead Compounds against Mycobacterium abscessus and Malaria Parasites . *ACS Infect Dis* (2023) doi:10.1021/ACSINFECDIS.2C00537/ASSET/IMAGES/MEDIUM/ID2C00537_0032.GIF.

2. Kollman, P. A. *et al.* Calculating structures and free energies of complex molecules: Combining molecular mechanics and continuum models. *Accounts of Chemical Research* **33**, 889–897 (2000).

3. Wang, E. *et al.* End-Point Binding Free Energy Calculation with MM/PBSA and MM/GBSA: Strategies and Applications in Drug Design. *Chemical Reviews* **119**, 9478–9508 (2019).

4. And, G. A. K., Friesner*, R. A., And, J. T.-R. & Jorgensen, W. L. Evaluation and Reparametrization of the OPLS-AA Force Field for Proteins via Comparison with Accurate Quantum Chemical Calculations on Peptides†. (2001) doi:10.1021/JP003919D.

5. Xiao, L., Diao, J., Greene, D., Wang, J. & Luo, R. A Continuum Poisson-Boltzmann Model for Membrane Channel Proteins. *Journal of Chemical Theory and Computation* (2017) doi:10.1021/acs.jctc.7b00382.

6. Botello-Smith, W. M. & Luo, R. Applications of MMPBSA to Membrane Proteins I: Efficient Numerical Solutions of Periodic Poisson-Boltzmann Equation. *Journal of Chemical Information and Modeling* **55**, 2187–2199 (2015).

7. Botello-Smith, W. M. *et al.* Numerical Poisson-Boltzmann model for continuum membrane systems. *Chemical Physics Letters* (2013) doi:10.1016/j.cplett.2012.10.081.

8. Wang, J., Cieplak, P. & Kollman, P. A. How Well Does a Restrained Electrostatic Potential (RESP) Model Perform in Calculating Conformational Energies of Organic and Biological Molecules? *Journal of Computational Chemistry* **21**, 1049–1074 (2000).

9. Hornak, V. *et al.* Comparison of multiple amber force fields and development of improved protein backbone parameters. *Proteins: Structure, Function and Genetics* vol. 65 712–725 Preprint at https://doi.org/10.1002/prot.21123 (2006).

10. Zhang, B. *et al.* Crystal Structures of Membrane Transporter MmpL3, an Anti-TB Drug Target. *Cell* (2019) doi:10.1016/j.cell.2019.01.003.
